# A multi‐cohort study of longitudinal and cross‐sectional Alzheimer's disease biomarkers in cognitively unimpaired older adults

**DOI:** 10.1002/alz.14492

**Published:** 2025-01-27

**Authors:** Long Xie, Sandhitsu R. Das, Yue Li, Laura E. M. Wisse, Emily McGrew, Xueying Lyu, Michael DiCalogero, Ujashi Shah, Ademola Ilesanmi, Amanda E. Denning, Chris A. Brown, Jesse Cohen, Lasya Sreepada, Mengjin Dong, Niyousha Sadeghpour, Pulkit Khandelwal, Ranjit Ittyerah, Sadhana Ravikumar, Shokufeh Sadaghiani, Stanislau Hrybouski, Robin de Flores, Eli Gibson, Paul A. Yushkevich, David A. Wolk, for the Alzheimer's Disease Neuroimaging Initiative

**Affiliations:** ^1^ Department of Digital Technology and Innovation Siemens Healthineers Princeton New Jersey USA; ^2^ Penn Image Computing and Science Laboratory (PICSL) Department of Radiology University of Pennsylvania Philadelphia Pennsylvania USA; ^3^ Department of Clinical Sciences Lund Lund University Lund Sweden; ^4^ Penn Memory Center University of Pennsylvania Philadelphia Pennsylvania USA; ^5^ Department of Neurology University of Pennsylvania Philadelphia Pennsylvania USA; ^6^ Université de Caen Normandie INSERM UMRS U1237 Caen France

**Keywords:** amyloid, biomarkers, disease progression, magnetic resonance imaging, neurodegeneration, normal aging, preclinical Alzheimer's disease, prediction, tau

## Abstract

**INTRODUCTION:**

The generalizability of neuroimaging and cognitive biomarkers in their sensitivity to detect preclinical Alzheimer's disease (AD) and power to predict progression in large, multisite cohorts remains unclear.

**METHOD:**

Longitudinal demographics, T1‐weighted magnetic resonance imaging (MRI), and cognitive scores of 3036 cognitively unimpaired (CU) older adults (amyloid beta [Aβ]‐negative/positive [A–/A+]: 1270/1558) were included. Cross‐sectional and longitudinal cognition and medial temporal lobe (MTL) structural measures were extracted. Cross‐sectional MTL tau burden (T) was computed from tau positron emission tomography (*N* = 1095).

**RESULTS:**

We found cross‐sectional tau and longitudinal structural biomarkers best separated A+ CU from A– CU. A–T+ CU had significantly faster neurodegeneration rate compared to A–T– CU. MTL tau was significantly correlated with MRI and cognitive biomarkers regardless of Aβ status. MTL tau, MRI, and cognition provided complementary information about disease progression.

**DISCUSSION:**

This large multisite study replicates prior findings in CU older adults, supporting the utility of neuroimaging and cognitive biomarkers in preclinical AD clinical trials and normal aging studies.

**Highlights:**

- We investigated neuroimaging and cognitive biomarkers in 3036 cognitively unimpaired (CU) participants.
- Medial temporal lobe (MTL) tau and longitudinal MTL atrophy best separate amyloid beta positive (A+) CU from amyloid beta negative (A–) CU.
- A– tau positive (T+) CU had a significantly faster neurodegeneration rate compared to A–T– CU.
- MTL tau correlated with structural magnetic resonance imaging (MRI) and cognition regardless of amyloid beta status.
- Combined baseline MTL tau, MRI, and cognition best predict Alzheimer's disease progression.

## 1 BACKGROUND

Recent breakthroughs in anti‐amyloid monoclonal antibodies have marked a new era in Alzheimer's disease (AD) care. These treatments slow down cognitive decline in symptomatic patients with mild cognitive impairment (MCI) and mild dementia due to AD.^1^, ^2^ Interventions are likely to be more effective if administered earlier in the disease course when cognitive symptoms are minimal or not present, that is, in the preclinical phase of AD.^3^ However, evaluating treatment efficacy in this population is challenging due to subtle and slow cognitive changes, necessitating thousands of participants and relatively long trials (e.g., 3–5 years). Therefore, developing effective biomarkers sensitive to disease progression in cognitively unimpaired (CU) individuals is crucial.

Prior studies have identified neuroimaging and cognitive measures sensitive to preclinical AD in relatively limited samples. While structural changes outside the medial temporal lobe (MTL), such as medial frontal areas and precuneus and cingulate,^4^, ^5^ have been reported, consistent structural differences in the MTL have been identified in preclinical AD likely due to its early involvement in AD pathology. However, recent findings suggest that the extent of tau pathology, especially in the neocortex, significantly influences cognitive decline, with MTL‐specific tau also increasing the risk of future decline.^6^ Indeed, these insights have been integrated into new biomarker‐driven diagnostic criteria.^7^ Nonetheless, as the MTL is among the earliest areas of tau pathology, we focus on this region specifically in the current work. Using 76 amyloid beta (Aβ) positive (A+) and 151 Aβ negative (A–) CU participants, our prior work^8^ found significantly faster longitudinal atrophy rates in the anterior and posterior hippocampus and Brodmann area 35 (BA35), which overlaps largely with the transentorhinal region.^9^ However, only a marginal difference was seen in BA35 cross‐sectionally. This is consistent with Koenig et al.,^10^ which reported no significant differences in hippocampal volume and entorhinal cortex (ERC) thickness between 115 preclinical AD and 192 age‐matched controls, possibly due to limited sample size.

Another set of studies investigated the correlation between MTL tau burden, magnetic resonance imaging (MRI)‐based structural atrophy, and cognition in CU individuals.^11^, ^12^, ^13^, ^14^ Berron et al. observed a significant correlation between tau burden and structural measures in the MTL in 79 A+ and 217 A– CU participants.^11^ Similarly, using a dataset of 36 A+ and 47 A– CU, Maass et al. reported ERC thickness was most closely associated with tau tracer uptake in ERC.^12^ A significant association between MTL tau burden and episodic memory was observed in both studies. However, conflicting findings emerged regarding the association between cross‐sectional MTL atrophy and memory performance, likely due to insufficient power. Berron et al.^11^ reported no significant association, while Maass et al.^12^ found a statistically significant effect. Interestingly, Maass et al. reported that the associations among tau, structural MRI, and memory remained significant in A– CU participants, suggesting a potential connection in “normal” aging. Replication is needed in a larger and broader sample.

In addition, studies have explored the prognostic value of baseline neuroimaging and cognitive biomarkers in CU individuals. Maass et al. reported a significant association between cross‐sectional tau burden or cross‐sectional ERC thickness and decline in episodic memory or longitudinal entorhinal atrophy.^12^ Previous studies have also shown that positivity in both Aβ and tau pathologies in CU older adults is associated with structural atrophy and global cognitive decline—including clinical progression to MCI.^6^, ^15^ However, none of the prior work directly compared or investigated the complementary information provided by tau positron emission tomography (PET), structural MRI, and cognitive biomarkers in predicting disease progression. Identifying the most predictive biomarkers will offer valuable insight for clinical practice.

While the above‐mentioned results support the potential clinical utility of tau PET, structural MRI, and cognitive measures in preclinical AD, their interpretability was constrained by the limited sample sizes. Hence, in this study, we pooled structural MRI, tau PET, and standardized cognitive test scores of 3036 CU individuals (1558 A+ and 1270 A–) from four AD studies to resolve the aforementioned discrepancies. Specifically, we investigated the differences of tau PET (cross‐sectional), structural MRI (both cross‐sectional and longitudinal), and cognitive (both cross‐sectional and longitudinal) biomarkers among subgroups of CU individuals defined by Aβ and tau status. The relationships among tau PET, structural MRI, and cognitive measures were examined. In addition, we tested the power of the optimal combination of baseline biomarkers at predicting future disease progression in both A+ and A– CU individuals. The MTL was the focus in this study because it is the first cortical site affected by AD.^9^ The overall goal was to provide stronger evidence for the utility of these biomarkers in identifying individuals who are most likely to benefit from early interventions and in evaluating outcomes of clinical trials.

## 2 METHODS

**FIGURE 1 alz14492-fig-0001:**
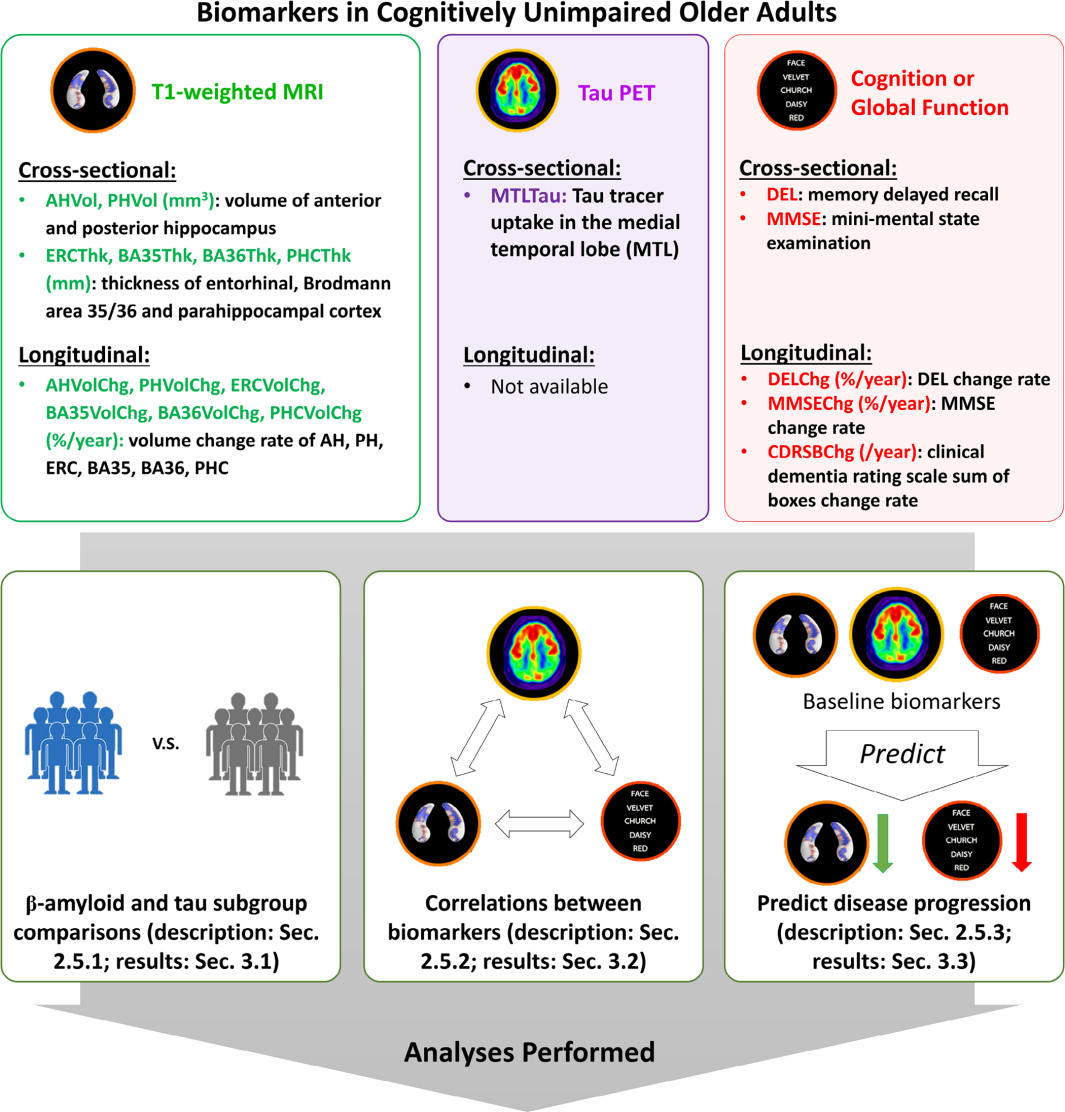
Schematic figure with the list of cross‐sectional and longitudinal neuroimaging and cognitive/global functional biomarkers used in this study (top) and the analyses performed (bottom). Longitudinal tau biomarkers were not analyzed due to limited longitudinal tau PET data. Structural MRI, cognition, and tau PET measures were color‐coded in green, red, and purple, respectively, for easier interpretation of the results in the other tables and figures. See section 2.5 for the details of statistical analyses performed. AH, anterior hippocampus; BA35/36, Brodmann area 35/36; ERC, entorhinal cortex; MRI, magnetic resonance imaging; PET, positron emission tomography; PH, posterior hippocampus; PHC, parahippocampal cortex

To maximize statistical power in the earliest phase of AD, we pooled demographic, neuroimaging, and cognitive data of 3036 CU individuals (Table 1) from publicly available studies, including the Alzheimer's Disease Neuroimaging Initiative (ADNI, adni.loni.usc.edu, ida.loni.usc.edu), the Anti‐Amyloid treatment in Asymptomatic AD study (A4, a4study.org, ida.loni.usc.edu),^16^ and the Harvard Aging Brain study (HABS, habs.mgh.harvard.edu),^17^ as well as the Aging Brain Cohort (ABC) study conducted by the University of Pennsylvania Alzheimer's Disease Research Center (ADRC, pennmemorycenter.org/research/open‐research‐studies/abc). Three categories of biomarkers were investigated, including structural biomarkers of the MTL extracted from longitudinal T1‐weighted MRI, tau biomarkers from cross‐sectional tau PET, and psychometric measures. The complete list of biomarkers and an overview of analyses performed are shown in Figure 1. Details of biomarker computation and statistical analyses are provided below.

**TABLE 1 alz14492-tbl-0001:** Basic characteristics, cross‐sectional, and longitudinal biomarkers of the CU individuals, and separately for Aβ negative (A–) and positive (A+) subgroups.

	All CU (3036)	A– CU (1270)	A+ CU (1558)	*p* (A– vs. A+)	Cohen *d* (95% CI)
**Basic characteristics**					
A4/HABS/ADNI/ABC	1770/276/729/261	535/201/424/110	1235/74/217/32	< 0.001	
Age (years)	71.6 (7.1)	71.3 (5.8)	72.6 (5.5)	< 0.001	0.23 [0.16, 0.31]
Education (years)	16.5 (2.7)	16.6 (2.7)	16.5 (2.8)		
Sex (M/F)	1225/1811	527/743	629/929		
Race (%)					
Asian	66 (2.2%)	31 (2.4%)	31 (2.0%)		
Black or African	211 (6.9%)	104 (8.2%)	58 (3.7%)		
Indian or Alaskan	9 (0.3%)	7 (0.6%)	2 (0.1%)		
White	2657 (87.5%)	1104 (86.9%)	1447 (92.9%)		
More than one	21 (0.7%)	13 (1.0%)	4 (0.3%)		
Missing	72 (2.4%)	11 (0.8%)	16 (1.0%)		
Ethnicity					
Hispanic/Latino	108 (3.6%)	57 (4.5%)	42 (2.7%)		
Not Hispanic/Latino	2857 (94.1%)	1206 (95.0%)	1501 (96.3%)		
Missing	71 (2.3%)	7 (0.6%)	15 (1.0%)		
*APOE* ɛ4 (NC/C)	1671/1197	959/279	649/884	< 0.001	
CDRSB	0.06 (0.19)	0.07 (0.20)	0.06 (0.17)		
**Cross‐sectional**					
MRI sample #	3036	1270	1558		
AHVol (mm^3^)	1726 (223)	1731 (219)	1726 (227)		0.02 [–0.05, 0.09]
PHVol (mm^3^)	1631 (159)	1640 (157)	1626 (160)	<0.05	0.09 [0.02, 0.16]
ERCThk (mm)	2.07 (0.16)	2.07 (0.15)	2.07 (0.16)		0.03 [–0.05, 0.10]
BA35Thk (mm)	2.34 (0.17)	2.35 (0.17)	2.33 (0.17)	<0.05	0.10 [0.02, 0.17]
BA36Thk (mm)	2.40 (0.20)	2.40 (0.20)	2.39 (0.21)		0.03 [–0.04, 0.11]
PHCThk (mm)	2.18 (0.15)	2.18 (0.14)	2.18 (0.15)		0.00 [–0.08, 0.07]
DEL sample #	3020	1263	1554		
DEL	12.9 (3.5)	13.1 (3.5)	12.7 (3.5)	<0.01	0.10 [0.02, 0.17]
MMSE sample #	3025	1265	1557		
MMSE	28.9 (1.2)	29.0 (1.1)	28.8 (1.3)	<0.001	0.20 [0.12, 0.27]
Tau PET sample #	1095	488	599		
MTLTau	1.13 (0.15)	1.09 (0.10)	1.16 (0.18)	<0.001	0.50 [0.38, 0.62]
**Longitudinal**					
MRI sample #	772	508	217		
Follow‐up year	3.20 (1.01)	3.29 (1.00)	3.13 (1.03)		
# of timepoints	3.04 (1.30)	3.06 (1.31)	3.15 (1.31)		
AHVolChg (%/yr)	−0.28 (0.44)	−0.23 (0.38)	−0.36 (0.47)	<0.001	0.32 [0.16, 0.48]
PHVolChg (%/yr)	−0.26 (0.39)	−0.21 (0.35)	−0.34 (0.45)	<0.001	0.33 [0.17, 0.49]
ERCVolChg (%/yr)	−0.51 (0.90)	−0.38 (0.82)	−0.73 (0.96)	<0.001	0.41 [0.25, 0.57]
BA35VolChg (%/yr)	−0.84 (0.87)	−0.71 (0.75)	−1.10 (1.05)	<0.001	0.46 [0.30, 0.62]
BA36VolChg (%/yr)	−0.60 (0.74)	−0.51 (0.64)	−0.79 (0.90)	<0.001	0.38 [0.22, 0.54]
PHCVolChg (%/yr)	−0.60 (0.61)	−0.52 (0.57)	−0.75 (0.65)	<0.001	0.37 [0.21. 0.53]
DEL sample #	864	580	245		
Follow‐up year	3.39 (0.96)	3.42 (0.97)	3.37 (0.96)		
# of timepoints	3.59 (1.13)	3.61 (1.17)	3.59 (1.06)		
DELChg (%/yr)	3.17 (12.14)	3.92 (12.34)	1.83 (11.83)	<0.05	0.17 [0.02, 0.32]
MMSE sample #	865	581	245		
Follow‐up year	3.39 (0.95)	3.42 (0.97)	3.37 (0.95)		
# of timepoints	3.81 (1.18)	3.82 (1.22)	3.84 (1.12)		
MMSEChg (%/yr)	−0.23 (2.15)	−0.20 (2.29)	−0.33 (1.80)		0.06 [−0.09, 0.21]
CDRSB sample #	881	594	249		
Follow‐up year	3.37 (0.97)	3.39 (0.98)	3.34 (0.97)		
# of timepoints	3.78 (1.19)	3.78 (1.23)	3.83 (1.13)		
CDRSBChg (/yr)	0.06 (0.20)	0.04 (0.19)	0.09 (0.21)	<0.001	0.28 [0.13, 0.43]

*Note*: Covariates for all analyses: age and sex. Additional covariates for cross‐sectional volume analyses: intracranial volume. Additional covariates for cross‐sectional and longitudinal cognitive measurements: years of education. Additional covariates for longitudinal analyses: follow‐up time. Maximum follow‐up time was set to 5.0 years. Only participants with at least 1 year of follow‐up data were included in the longitudinal analysis. Statistical analysis between A– and A+ CU was performed, and *p* value and Cohen *d* were reported. Bar plots of all biomarkers were shown in Figure S2 in supporting information. See Figure 1 for biomarker abbreviations. Structural MRI, cognition, and tau PET measures were color‐coded in green, red, and purple, respectively, for easier interpretation in all tables and figures.

Abbreviations: #, number; A4, Anti‐Amyloid treatment in Asymptomatic AD study; A+/A–, amyloid beta positive/negative; Aβ, amyloid beta; ABC, Aging Brain Cohort; ADNI, Alzheimer's Disease Neuroimaging Initiative; *APOE*, apolipoprotein E; AUC, area under the curve; CI, confidence interval; CDRSB, Clinical Dementia Rating Sum of Boxes; CU, cognitively unimpaired; DEL, memory delayed recall; F, female; HABS, Harvard Aging Brain Study; M, male; MMSE, Mini‐Mental State Examination; MRI, magnetic resonance imaging; MTL, medial temporal lobe; NC/C, *APOE* ɛ4 non‐carrier/carrier; PET, positron emission tomography; PHCVol, parahippocampal cortex volume; yr, year.

### 2.1 Participants

Basic characteristics of the multisite dataset of CU individuals included in this study are summarized in Table 1.

**RESEARCH IN CONTEXT**

1. **Systematic review**: Traditional sources (e.g., PubMed), meeting abstracts, and presentations were used to perform a literature review to identify relevant prior research studies. There have been several recent publications investigating neuroimaging and cognitive biomarkers in cognitively unimpaired (CU) older adults, which have been appropriately cited.
2. **Interpretation**: Our multisite study, involving neuroimaging and cognitive biomarkers across 3036 CU individuals, demonstrated the utility of multimodal biomarkers in characterizing preclinical Alzheimer's disease (AD) and normal aging. These biomarkers may identify individuals most likely to benefit from interventions in the near term, as well as a potential means to monitor outcomes.
3. **Future directions**: We aim to incorporate longitudinal tau positron emission tomography and plasma biomarkers to further understand preclinical AD and normal aging, examine tau and structural measures in additional AD‐related brain areas for a more comprehensive picture of the disease, and enhance participant diversity to minimize biases and improve the applicability of our findings.

#### 2.1.1 ADNI

A total of 729 CU older adults with demographics, apolipoprotein E (*APOE*) ɛ4 genotype, longitudinal 3 Tesla (3T) T1‐weighted (T1w) MRI, longitudinal cognitive scores from ADNI GO, ADNI 2, and ADNI 3 were included in this study (adni.loni.usc.edu). CU diagnosis criteria include a score of 0 on the Clinical Dementia Rating (CDR) scale, a score of > 24 on the Mini‐Mental State Examination (MMSE) and a score above the age and education‐adjusted cutoffs on the Delayed Recall of the Logical Memory Story test^18^ (detailed cut‐off values are available at: adni.loni.usc.edu/help‐faqs/adni‐documentation). Among them, 393 participants had cross‐sectional tau (^18^F‐Flortaucipir) PET scans available. The Aβ status (A–/A+) in a subset of participants was publicly available as processed data in the ADNI database using a standard pipeline, derived from Aβ PET scans, described in detail at https://adni.loni.usc.edu/wp‐content/themes/freshnews‐dev‐v2/documents/pet/ADNI%20Centiloids%20Final.pdf. Thresholds of ^18^F‐Florbetapir standardized uptake value ratio (SUVR) ≥ 1.11 (Centiloid = 20) and ^18^F‐Florbetaben SUVR ≥ 1.08 (Centiloid = 18) were applied, as validated by Royse et al.^19^ Only Aβ PET scans within 1 year of the corresponding T1‐weighted MRI scan were considered. A total of 424 and 217 CU participants were A– and A+, respectively. The ADNI study was approved by the institutional review boards (IRB) of all the participating institutions. Informed written consent was provided by all participants at each site.^16^, [Table alz14492-tbl-0001]

#### 2.1.2 A4

A total of 1770 CU participants were included from the A4 study (a4study.org). CU participants must have an MMSE score between 25 and 30 as well as a CDR score of 0.^16^ Basic demographic and *APOE* ɛ4 genotype information were available in the released meta data. Only cross‐sectional MRI scans were available, and no tau PET scans were released at the time of the original data request (2021). ^18^F‐Flortaucipir PET scans were made publicly available after the completion of the neuroimaging processing of the current study. Hence, the publicly available regional SUVR spreadsheet (TAUSUVR.csv from ida.loni.usc.edu, inquiry date: August 9, 2024) was used instead (see section 2.2.4). Aβ status was determined using a global neocortical ^18^F‐Florbetapir PET SUVR threshold of ≥ 1.10, with the whole cerebellum as the reference region, as described in Insel et al.^16^ Because all screening procedures, including Aβ PET and MRI, need to be completed in 90 days according to the A4 study design (publicly available at cdn.clinicaltrials.gov/large‐docs/57/NCT02008357/Prot_000.pdf), we did not exclude A4 participants due to long time interval between Aβ PET and MRI scans. A total of 535 participants were A– and 1235 participants were A+. The A4 study was approved by the IRBs of all the participating institutions. Informed written consent was provided by all participants at each site.^17^, [Fig alz14492-fig-0001]

#### 2.1.3 HABS

HABS data (release 2.0) were obtained in December 2021 via habs.mgh.harvard.edu. Demographics, *APOE* ɛ4 genotype, longitudinal cognition, and T1‐weighted MRI from 276 CU participants were included. CU participants needed to score 0 on CDR, > 25 on MMSE, above age‐adjusted cutoffs on the Delayed Recall of the Logical Memory Story (the same as ADNI), and < 11 on the Geriatric Depression Scale.^17^ ^18^F‐Flortaucipir PET scans were available for 185 participants. In a subset of participants, Aβ positivity was available publicly in the processed HABS data. It was determined using a distribution volume ratio (DVR) threshold of ≥ 1.2 (which corresponds to 26 Centiloid). This DVR was computed from a composite of neocortical regions (frontal, lateral temporal, and retrosplenial cortices) with cerebellar gray matter as the reference region.^6^ Only Aβ PET scans within 1 year of the corresponding T1‐weighted MRI scan were considered and used to compute Aβ positivity. Two hundred one and 74 participants were A– and A+, respectively. The HABS protocol received approval from the Mass General Brigham IRB, and participants gave written informed consent before any procedures were performed.

#### 2.1.4 ABC

A total of 261 CU participants from the ABC study were included. Demographics, *APOE* ɛ4 genotype, and 3T T1w MRI were available in all participants. CU diagnosis is based on detailed psychometric testing, obtaining informant‐ and patient‐based history, and a detailed neurological and medical examination as prescribed by the Uniform Data Set 3.0 (UDS 3.0). Designation of CU is then made based on an annual consensus conference reviewing these materials without strict psychometric cutoffs. ^18^F‐Flortaucipir PET scans were acquired in 69 participants. Aβ status was derived in a subset of the participants from ^18^F‐Florbetaben or ^18^F‐Florbetapir PET scans, applying routine clinical visual reading assessment. Prior work has generally supported a high concordance between visual reading and quantitative designations of Aβ status.^20^, ^21^ Only Aβ PET scans within 1 year of the corresponding T1‐weighted MRI scan were considered and used to compute Aβ positivity, yielding 110 A– CU and 32 A+ CU. Written informed consent was provided by all participants under a protocol approved by the University of Pennsylvania IRB.

#### 2.1.5 Statement on human studies

The current study was performed in accordance with the ethical standards as laid down in the 1964 Declaration of Helsinki and its later amendments or comparable ethical standards.

### 2.2 Data acquisition and processing of neuroimaging biomarkers

#### 2.2.1 Imaging data acquisition

In ADNI, the 3T T1w MRI, tau PET (^18^F‐Flortaucipir), and Aβ PET (^18^F‐Florbetaben or ^18^F‐Florbetapir) scans were acquired from different scanners at multiple sites. The preprocessed tau PET scan (“AV1451 Coreg, Avg, Std Img and Vox Siz, Uniform Resolution”) was used. Up‐to‐date information about MRI imaging protocols can be found at adni.loni.usc.edu/methods/mri‐tool/mri‐analysis. Details of PET acquisition and processing are available at adni.loni.usc.edu/methods/pet‐analysis‐method/pet‐analysis.

For HABS, tau PET (^18^F‐Flortaucipir), amyloid PET (Pittsburgh Compound B [PiB]), and 3T T1w MRI scans were acquired at the Massachusetts General Hospital in Massachusetts, USA. Preprocessed tau PET scans were available for download and used in this study. Complete MRI and PET acquisition and processing can be found at https://habs.mgh.harvard.edu/wp‐content/uploads/2020/08/HABS_ImageDataRelease_2.0_Notes.pdf and on the HABS website (habs.mgh.harvard.edu).

The A4 study's 3T T1w MRI, tau PET (^18^F‐Flortaucipir), and Aβ PET (^18^F‐Florbetapir) scans were acquired from different scanners at multiple sites. Details of neuroimaging acquisition and processing can be found in Sperling et al.^3^

All scans for the ABC study were acquired at the University of Pennsylvania ADRC. A whole‐brain 3T T1w 3D magnetization prepared rapid gradient echo (MPRAGE) sequence (repetition time: 2400 ms; echo time: 2.24 ms; inversion time: 1060 ms; flip angle: 8°; field of view: 256 × 240 × 167 mm^3^; voxel size: 0.8 × 0.8 × 0.8 mm^3^) was used to acquire structural MRI images. Aβ PET ^18^F‐Florbetaben (or ^18^F‐Florbetapir) protocol used an injection of 8.1 mCI of tracer (10 mCi for ^18^F‐Florbetapir) and a 20 minute brain scan (4 frames of 5 minute duration) after a 90 minute (50 minutes for ^18^F‐Florbetapir) uptake phase. A 30 minute tau PET brain scan (6 frames of 5 minutes’ duration) was performed 75 minutes after the injection of ≈ 10 mCi of the ^18^F‐Flortaucipir tracer.

#### 2.2.2 Cross‐sectional structural MRI biomarkers of the MTL

Using a tailored segmentation pipeline, Automatic Segmentation of Hippocampal Subfields‐T1 (ASHS‐T1, https://sites.google.com/view/ashs‐dox/), subregions of the MTL were automatically segmented in the earliest MRI scan (for the analyses not involving tau measures) as well as the MRI scan that was most proximal in time of acquisition to the corresponding tau PET scan (for analyses involving tau measures, referred to as the tau‐anchored MRI scan). The subregions include anterior/posterior hippocampus (AH/PH), ERC, BA35, and Brodmann area 36 (BA36), and parahippocampal cortex (PHC). ASHS‐T1 explicitly accounts for the confound of dura mater in the segmentation, overcoming the limitations of conventional approaches optimized for whole‐brain analysis^22^ and has been validated to perform accurate MTL subregion segmentation from in vivo T1w MRI.^23^ A summary region of interest (ROI) measurement was extracted for each subregion: for AH and PH, volume was computed directly from the automatic segmentation, while for the MTL cortical subregions (ERC, BA35, BA36, and PHC) median cortical thickness was computed by applying a graph‐based multi‐template thickness analysis pipeline^24^ to the ASHS‐T1 segmentation.

In addition, regional thickness maps of the MTL with pointwise correspondence between participants were generated from the ASHS‐T1 segmentations using the “cortical reconstruction for ASHS (CRASHS)” surface‐based pipeline,^25^ detailed in Material S2 in supporting information. Figure S1a in supporting information shows the average regional thickness maps of the left and right MTL overlaid on a geometric template that represents the average inflated MTL surface. Statistical analysis can be performed at each point on the surface to investigate regional disease effects in MTL.

Intracranial volume (ICV), which was used as a covariate in the analysis (see section 2.5), was computed from the cross‐sectional structural MRI using an in‐house ICV segmentation software implemented using the ASHS codebase.^23^

#### 2.2.3 Longitudinal structural MRI biomarkers of the MTL

Using a previously validated pipeline,^8^ the longitudinal annualized atrophy rate of each subregion was extracted. In brief, the Automatic Longitudinal Hippocampal Atrophy (ALOHA) pipeline^26^ was applied to all pairs of baseline and prospective follow‐up longitudinal MRI scans, along with the baseline subregion segmentation mask generated by ASHS‐T1 in section 2.2.2, to estimate the change in volume in an unbiased manner based on symmetric diffeomorphic registration.^27^ The baseline scan was set to the earliest MRI scan, or the tau‐anchored MRI scan, depending on whether the analysis involved tau measures. Change measures between the baseline scan and each follow‐up scan were used to estimate follow‐up volumes. A linear model was fit to the estimated volumes of all the timepoints to derive the volume change rate for each MTL subregion. Dividing the change rate by the baseline volume yields a measure of relative volume atrophy rate in percentage per year. Bilateral measurements were averaged to improve reliability in the ROI level analysis.

In addition to extracting longitudinal measurements for each subregion (ROI level), we computed the longitudinal regional atrophy rate maps using an established pipeline that combines skeletonization and deformation‐based morphometry, detailed in Yushkevich et al.^25^ In brief, the pipeline uses deformable registration similar to ALOHA but with additional geometric regularization to estimate the change in thickness between the baseline scan and each follow‐up scan at each point on the MTL gray matter surface. Given these thickness change estimates for follow‐up scans for a participant, linear regression analyses were performed to estimate the longitudinal atrophy rate on each surface point, similar to that of the ROI analyses, which results in a map of atrophy rate measures for the left and right MTL of each participant. The average bilateral regional MTL atrophy rate maps of A– and A+ CU participants are shown in Figure S1b.

Longitudinal MRI scans within 5 years from baseline MRI prospectively were included in the current study. Participants with only follow‐up scans within 1 year (i.e., no follow‐up between 1 and 5 years from baseline) were excluded, as abnormal longitudinal atrophy rate within a year may be too small to detect in the earliest phase of the disease.

#### 2.2.4 Cross‐sectional tau burden in the MTL

Cross‐sectional ^18^F‐Flortaucipir tau PET scans from 655 participants were available in ADNI, HABS, and ABC for the extraction of tau burden measures. We first converted the images to SUVR maps using tau PET signal in cerebellar gray matter as the reference region. Rigid registration was performed between the tau SUVR map and tau‐anchored MRI scan using Greedy registration software^28^ (github.com/pyushkevich/greedy). Then the tau SUVR map was transformed and resampled to the space of the tau‐anchored MRI scan. The mean tau PET signal in the union of ERC and BA35 labels was computed and served as a tau burden measurement in the MTL. Bilateral measurements were averaged for the ROI analysis, referred to as MTLTau.

Cross‐sectional ^18^F‐Flortaucipir tau PET scans with derived regional SUVR measurements from the A4 study were made publicly available after the completion of neuroimaging processing of the current study. Consequently, instead of deriving MTLTau measures from tau PET scans as done in the other three studies, we used bilateral tau SUVR in the ERC (mean_ctx_lh_entorhinal and mean_ctx_rh_entorhinal) from the TAUSUVR.csv spreadsheet publicly available at ida.loni.usc.edu (inquiry date: August 9, 2024) as indicators of MTL tau burden. The bilateral measures were averaged to compute MTLTau for each subject. In total, cross‐sectional MTLTau measures for 440 CU participants from the A4 study were included, resulting in a total of 1095 participants with cross‐sectional MTLTau measures. Data harmonization was performed to remove potential confounds due to methodological differences between the A4 study and the other studies, described in detail in section 2.4.

To investigate tau subgroup effects, we determined the tau positivity (T– and T+) of each CU individual, thresholding the harmonized MTLTau measure. A two‐component Gaussian mixture model^29^ was fitted to the MTLTau of all CU individuals, and the threshold was computed as the mean plus two times the standard deviation (SD) of the Gaussian component with the lower mean, the same as prior studies.^30^, ^31^, ^32^, ^33^ The threshold was an SUVR of 1.27, which resulted in 163 T+ and 932 T– CU participants.

#### 2.2.5 Quality control

Detailed quality control steps were performed at all stages of image processing to ensure acceptable quality of biomarker measures.

For quality control for MRI processing, all longitudinal MRI scans were visually checked for severe motion artifact or blurring. Visual inspection was also performed on all ASHS‐T1 segmentations. A subset of individuals with small errors were identified and were excluded. Both the multi‐template thickness analysis pipeline (used to extract cross‐sectional thickness of the MTL cortical regions) and CRASHS (used to extract regional thickness maps of the MTL) provide quality of fit measures that were used to remove cases with poor measurement quality.

In addition, the quality of longitudinal alignment between MRI scans was checked using the workflow described in Xie et al.^8^ In the workflow, pairs with poor normalized correlation after alignment were identified and visually inspected to ensure quality. Pairs of MRIs that were marked as failed were excluded from the longitudinal change rate calculation.

For quality control for tau PET processing, tau PET scans from ADNI, HABS, and ABC were available for analysis and the scans were visually checked to ensure correct reconstruction. Registration between tau PET and MRI was checked visually to ensure good alignment, which is crucial for tau burden measures in the MTL.

### 2.3 Data acquisition and processing of cognitive biomarkers

Standard cognitive assessments that were available in all four datasets were used in this study, including MMSE, and Clinical Dementia Rating scale Sum of Boxes (CDRSB) scores. Memory delayed recall (DEL) was accessed using the Wechsler Memory Scale Logical Memory test^18^ in ADNI, HABS, and A4. On the other hand, the ABC study used the Craft Story test available in the UDS 3.0^34^ to assess delayed recall function. Both tests applied similar procedures but differed in story content. To eliminate potential differences introduced by the test methodology, a harmonized story recall measure was used as described in section 2.4.

The longitudinal rate of cognitive change was computed for each measurement using the same linear modeling technique as the one used to compute the longitudinal structural atrophy rate in MRI (see section 2.2.3). One difference was that absolute change rate, rather than relative, was computed for longitudinal CDRSB change, as computing relative change was not feasible because most participants had a baseline CDRSB of 0. To be consistent with the longitudinal structural MRI measures, the timeframe of cognitive measures was set the same, that is, maximum follow‐up time was 5 years and participants with only 1 year of follow‐up measures were excluded.

### 2.4 Harmonization of biomarkers

Data harmonization has been demonstrated^35^ to benefit biomarker studies when working with data from multiple sources to account for potential differences in data acquisition and quantification. In this current study, we applied NeuroCombat (github.com/Jfortin1/neuroCombat_Rpackage) to harmonize all MRI‐based structural biomarkers and MTL tau burden measure with the dataset set as the batch variable. Because MMSE and CDRSB are established and well‐adapted standard cognitive assessment techniques, the raw measurements were used. Cross‐sectional and longitudinal DEL measures were harmonized because slightly different cognitive tests were used in ABC to assess delayed recall.

### 2.5 Experimental design and statistical analysis

Experiments were conducted to investigate differences of biomarkers between Aβ (A) and tau (T) subgroups of CU individuals; correlations between tau PET, structural, and cognitive biomarkers in the earliest stage of AD; and the predictive power of the combination of baseline biomarkers in disease progression. Statistical analyses were conducted in R 4.2.2 (www.r‐project.org) except for regional MTL analyses, which were conducted using mesh_glm from the CM‐Rep package (github.com/pyushkevich/cmrep). All statistical tests were two sided.

#### 2.5.1 Group comparison analysis

Basic characteristics and biomarkers were compared between A subgroups (A+ CU vs. A– CU, results in Table 1 with bar plots in Figure S2 in supporting information) and between A and T subgroups (A–T+ CU vs. A–T– CU, A+T– CU vs. A–T– CU, A+T+ CU vs. A–T– CU, and A+T+ CU vs. A+T– CU, results in Table 2 with bar plots in Figure S3 in supporting information). Independent two‐sample *t* tests were used to test mean group differences in age, years of education, and baseline CDRSB. χ^2^ tests were used to test for the presence of significant group differences in the proportion of source dataset, sex, *APOE* ɛ4 genotype, race, and ethnicity. General linear model (GLM) analyses were performed for all cross‐sectional and longitudinal biomarkers. Biomarker of interest was set as the dependent variable; group membership was the factor of interest, and age and sex were the nuisance covariates. ICV was included as an additional covariate for cross‐sectional volume measurements and follow‐up time was used as an additional covariate for longitudinal measurements. Years of education was included as an additional covariate for cognitive measurements. *APOE* ɛ4 genotype was not included as a covariate, but the results did not differ significantly with it in the models. The effect size of each A– CU versus A+ CU comparison was estimated using Cohen *d*. Meta‐analysis using the meta package in R was performed to test statistical differences between Cohen *d* values.

**TABLE 2:** Basic characteristics, cross‐sectional, and longitudinal biomarkers of the subgroups of CU individuals defined by Aβ (A+/A–) and tau (T+/T–) status.

	A–T– CU (462)	A‐T+ CU (26)	A+T– CU (462)	A+T+ CU (137)
**Basic characteristics**				
A4/HABS/ADNI/ABC	54/113/244/51	0/15/10/1	306/37/107/12	80/20/32/5
Age (years)	72.0 (6.5)	80.1 (6.2)^***^	73.3 (6.0)^***^	74.4 (5.8)^***^
Education (years)	16.5 (2.7)	16.5 (3.4)	16.2 (2.8)	16.4 (2.3)
Sex (M/F)	188/274	19/7^**^	198/264	52/85
Race (%)				
Asian	10 (2.2%)	0 (0.0%)	20 (4.3%)	2 (1.5%)
Black or African	43 (9.3%)	3 (11.5%)	22 (4.8%)	5 (3.6%)
Indian or Alaskan	3 (0.6%)	0 (0.0%)	0 (0.0%)	0 (0.0%)
White	396 (85.7%)	22 (84.6%)	415 (89.8%)	127 (92.7%)
More than one	7 (1.5%)	0 (0.0%)	0 (0.0%)	2 (1.5%)
Missing	3 (0.6%)	1 (3.8%)	5 (1.1%)	1 (0.7%)
Ethnicity				
Hispanic/Latino	22 (4.8%)	0 (0.0%)	6 (1.3%)	5 (3.6%)
Not Hispanic/Latino	439 (95.0%)	26 (100.0%)	448 (97.0%)	131 (95.6%)
Missing	1 (0.2%)	0 (0.0%)	8 (1.7%)	1 (0.7%)
*APOE* ɛ4 (NC/C)	336/112	25/1	221/234	34/95^***^
CDRSB	0.07 (0.23)	0.17 (0.28)^*^	0.07 (0.21)	0.14 (0.40)^*^
**Cross‐sectional**				
MRI sample #	462	26	462	137
AHVol (mm^3^)	1718 (208)	1757 (221)	1736 (228)	1680 (235)
PHVol (mm^3^)	1633 (152)	1587 (174)	1637 (157)	1554 (155)^***^
ERCThk (mm)	2.08 (0.14)	2.01 (0.14)^*^	2.08 (0.15)	2.01 (0.17)^***^
BA35Thk (mm)	2.34 (0.15)	2.36 (0.13)	2.34 (0.17)	2.28 (0.20)^***^
BA36Thk (mm)	2.40 (0.19)	2.31 (0.19)^*^	2.40 (0.19)	2.34 (0.22)^***^
PHCThk (mm)	2.17 (0.14)	2.24 (0.14)	2.17 (0.15)	2.16 (0.16)
DEL sample #	460	26	462	135
DEL	13.9 (3.3)	12.8 (4.0)	13.6 (3.6)	12.1 (3.6)^***^
MMSE sample #	461	26	462	136
MMSE	29.1 (1.0)	29.0 (1.3)	28.8 (1.3)^***^	28.5 (1.4)^***^
Tau PET sample #	462	26	462	63
MTLTau	1.08 (0.09)	1.33 (0.08)^***^	1.09 (0.10)	1.41 (0.14)^***^
**Longitudinal**				
MRI sample #	232	15	83	34
Follow‐up year	2.64 (0.84)	3.02 (0.82)	2.40 (0.78)	2.31 (0.56)
AHVolChg (%/yr)	−0.24 (0.39)	−0.21 (0.43)	−0.30 (0.42)	−0.69 (0.56)^***^
PHVolChg (%/yr)	−0.29 (0.45)	−0.29 (0.47)	−0.27 (0.41)	−0.59 (0.62)^***^
ERCVolChg (%/yr)	−0.36 (0.96)	−0.98 (1.07)^*^	−0.41 (0.98)	−1.11 (1.30)^***^
BA35VolChg (%/yr)	−0.77 (0.85)	−1.28 (1.31)^*^	−0.88 (0.86)	−1.66 (1.53)^***^
BA36VolChg (%/yr)	−0.57 (0.71)	−0.62 (1.32)	−0.66 (0.79)	−1.43 (1.44)^***^
PHCVolChg (%/yr)	−0.59 (0.64)	−0.81 (1.02)	−0.58 (0.69)	−0.93 (0.88)^**^
DEL sample #	283	17	105	43
Follow‐up year	2.87 (0.99)	2.71 (0.89)	2.89 (1.02)	2.55 (0.82)
DELChg (%/yr)	4.06 (14.11)	1.69 (15.47)	4.01 (17.56)	−1.15 (14.66)^*^
MMSE sample #	285	17	105	43
Follow‐up year	2.86 (0.98)	2.59 (0.83)	2.87 (1.01)	2.55 (0.82)
MMSEChg (%/yr)	−0.37 (3.03)	−1.31 (3.51)	−0.48 (2.06)	−0.63 (2.88)
CDRSB sample #	298	16	107	44
Follow‐up year	2.84 (0.97)	2.69 (0.91)	2.85 (1.00)	2.63 (0.85)
CDRSBChg (/yr)	0.02 (0.13)	0.05 (0.39)	0.06 (0.20)^*^	0.20 (0.29)^***^

*Note*: *, *p* < 0.05; **, *p* < 0.01; ***, *p* < 0.001, compared to A–T– CU. Covariates for statistical analyses: age and sex. Additional covariates for cross‐sectional volume analyses: intracranial volume. Additional covariates for cross‐sectional and longitudinal cognitive measurements: years of education. Additional covariates for longitudinal analyses: follow‐up time. Maximum follow‐up time was set to 5.0 years. Only participants with at least 1 year of follow‐up data were included in the longitudinal analysis. The sample size is smaller than that in Table 1 limited by the number of tau PET scans available. Bar plots of all biomarkers were shown in Figure S4 in supporting information. See Figure 1 for biomarker abbreviations. Structural MRI, cognition, and tau PET measures were color‐coded in green, red, and purple, respectively, for easier interpretation in all tables and figures.

Abbreviations: #, number; A4, Anti‐Amyloid treatment in Asymptomatic AD study; A+/A–, amyloid beta positive/negative; Aβ, amyloid beta; ABC, Aging Brain Cohort; ADNI, Alzheimer's Disease Neuroimaging Initiative; *APOE*, apolipoprotein E; CDRSB, Clinical Dementia Rating Sum of Boxes; CU, cognitively unimpaired; DEL, memory delayed recall; F, female; HABS, Harvard Aging Brain Study; M, male; MMSE, Mini‐Mental State Examination; MRI, magnetic resonance imaging; MTL, medial temporal lobe; NC/C, *APOE* ɛ4 non‐carrier/carrier; PET, positron emission tomography; PHCVol, parahippocampal cortex volume; T+/T–, tau positive/negative; yr, year.

**FIGURE 2:**
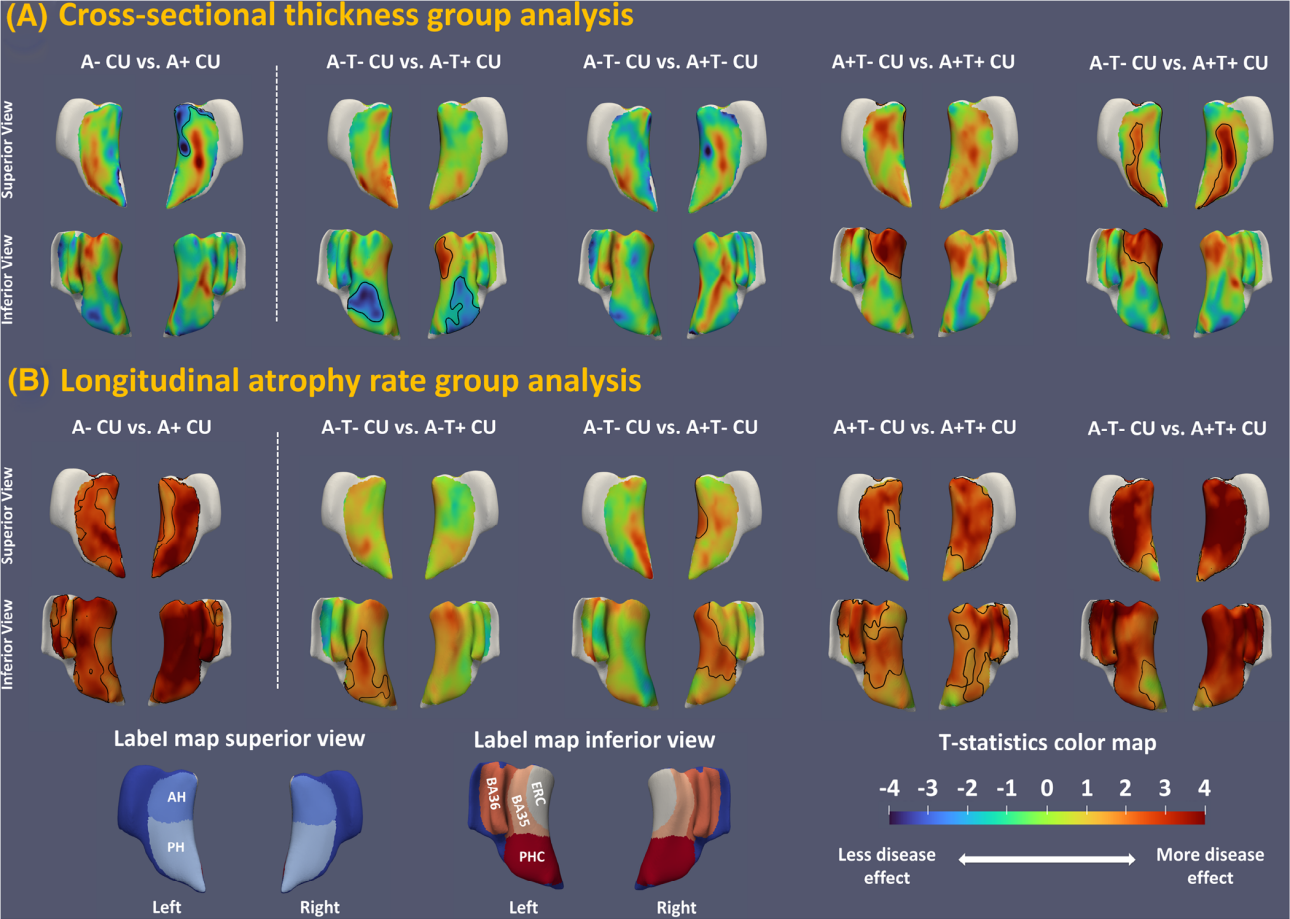
Two views of the statistical maps comparing cross‐sectional thickness (A) and longitudinal atrophy rate (B) between Aβ and tau subgroups of CU older adults specified above respective maps. Analyses of subgroups defined by Aβ alone are shown to the left of the white dashed line. Analyses of subgroups defined by both Aβ and tau are shown to the right of the white dashed line. Each statistical map plots the *t* statistics associated with the null hypothesis that structural measurement (either cross‐sectional thickness or longitudinal atrophy rate) at a given location in the MTL is equal between the two groups. Red colored *t* scores represent stronger disease effect (thinner thickness in cross‐sectional analyses and faster atrophy rate in longitudinal analyses), while blue colored *t* scores represent less disease effect. Clusters highlighted by black contours are statistically significant at *p* < 0.05 after correcting for multiple comparisons using cluster‐level FWER permutation test approach with 1000 permutations and cluster threshold is uncorrected *p* < 0.05 for thickness and *p *< 0.01 for longitudinal atrophy rate. A+/A–, amyloid beta positive/negative; Aβ, amyloid beta; AH, anterior hippocampus; BA35/36, Brodmann area 35/36; CU, cognitively unimpaired; ERC, entorhinal cortex; FWER, family‐wise error rate; MTL, medial temporal lobe; PH, posterior hippocampus; PHC, parahippocampal cortex; T+/T–, tau positive/negative

For MTL regional analysis, GLM analysis was performed at each surface point of the regional thickness or atrophy rate map with the same set up as the ROI analysis. Cluster‐level family‐wise error rate (FWER)^36^ with 1000 permutations was used to correct for multiple comparisons. Clusters were defined using uncorrected *p* < 0.05 for cross‐sectional thickness maps and uncorrected *p* < 0.01 for longitudinal atrophy rate maps. Analysis results are reported in Figure 2.

#### 2.5.2 Correlation analysis between biomarkers

Partial correlation analyses with age and sex as covariates were performed between MTLTau and each of the cross‐sectional and longitudinal MRI‐based structure (at the ROI level) and cognitive measures in A– CU and A+ CU subgroups separately. Separate models were performed in Aβ subgroups because we treat these subgroups as reflecting separate categories, that is, A+ individuals as being on the AD continuum while A– individuals are not.^7^, ^37^ The implications of correlations between biomarkers may differ in these contexts. Years of education was included as an additional covariate when cognitive measures were involved, and years of follow‐up time was a covariate for longitudinal measures. Partial correlation analyses, with age, sex, education, and ICV as covariates, were also performed between cross‐sectional structural measures and cognitive ones. Instead of performing analyses for all subregions, we selected cross‐sectional posterior hippocampal volume (PHVol) only as it consistently showed disease effect in the current and prior studies.^8^, ^23^ Results are reported in Table 3 with scatter plots in Figure S4 in supporting information. A post hoc mediation analysis was conducted using the “mediation” package in R to further investigate whether the significant correlation between MTLTau and longitudinal CDRSBChg was mediated by the cross‐sectional posterior hippocampal volume in the A+ CU subgroup (Figure S5 in supporting information). Measurements were adjusted for age and sex before the analysis.

**TABLE 3 alz14492-tbl-0003:** Partial correlation between tau, structural MRI, and cognition biomarkers. Section (a) shows correlations with MTLTau and section (b) shows correlations with PHVol.

	A– CU	A+ CU
**(a) Correlation with cross‐sectional MTLTau**		
**Cross‐sectional**		
MRI sample #	488	599
AHVol (mm^3^)^*^	ρ = –0.01, *p* > 0.1	ρ = –0.11, *p* = 0.009
PHVol (mm^3^)	ρ = –0.15, *p* < 0.001	ρ = –0.26, *p* < 0.001
ERCThk (mm)	ρ = –0.13, *p* = 0.003	ρ = –0.15, *p* < 0.001
BA35Thk (mm)	ρ = –0.06, *p* > 0.1	ρ = –0.13, *p* = 0.002
BA36Thk (mm)	ρ = –0.13, *p* = 0.003	ρ = –0.14, *p* < 0.001
PHCThk (mm)	ρ = –0.01, *p* > 0.1	ρ = 0.00, *p* > 0.1
DEL sample #	486	599
DEL	ρ = –0.13, *p* = 0.004	ρ = –0.18, *p* < 0.001
MMSE sample #	487	599
MMSE	ρ = –0.02, *p* > 0.1	ρ = –0.07, *p* > 0.1
**Longitudinal**		
MRI sample #	247	117
AHVolChg (%/year)	ρ = –0.17, *p* = 0.006	ρ = –0.37, *p* < 0.001
PHVolChg (%/year)	ρ = –0.16, *p* = 0.011	ρ = –0.28, *p* = 0.002
ERCVolChg (%/year)	ρ = –0.16, *p* = 0.011	ρ = –0.33, *p* < 0.001
BA35VolChg (%/year)	ρ = –0.24, *p* < 0.001	ρ = –0.38, *p* < 0.001
BA36VolChg (%/year)^*^	ρ = –0.15, *p* = 0.020	ρ = –0.39, *p* < 0.001
PHCVolChg (%/year)	ρ = –0.11, *p* = 0.079	ρ = –0.29, *p* = 0.002
DEL sample #	300	148
DELChg (%/year)	ρ = –0.04, *p* > 0.1	ρ = –0.15, *p* = 0.078
MMSE sample #	302	148
MMSEChg (%/year)	ρ = –0.07, *p* > 0.1	ρ = –0.03, *p* > 0.1
CDRSB sample #	314	151
CDRSBChg (/year)	ρ = 0.15, *p* = 0.009	ρ = 0.30, *p* < 0.001
**(b) Correlation with cross‐sectional posterior hippocampal volume (PHVol in mm^3^)**		
**Cross‐sectional**		
Tau PET sample #	488	599
MTLTau	ρ = –0.15, *p* < 0.001	ρ = –0.26, *p* < 0.001
MMSE sample #	1265	1557
MMSE	ρ = 0.01, *p* > 0.1	ρ = 0.01, *p* > 0.1
DEL sample #	1263	1554
DEL^*^	ρ = 0.00, *p* > 0.1	ρ = 0.08, *p* = 0.001
**Longitudinal**		
DEL Sample #	580	245
DELChg (%/year)	ρ = 0.09, *p* = 0.03	ρ = 0.15, *p* = 0.018
MMSE sample #	581	245
MMSEChg (%/year)^*^	ρ = –0.06, *p* > 0.1	ρ = 0.15, *p* = 0.015
CDRSB sample #	592	249
CDRSBChg (/year)^*^	ρ = –0.02, *p* > 0.1	ρ = ‐0.19, *p* = 0.002

*Note*: Orange background highlights significant correlations with *p* < 0.01 and *ρ* > 0.1. Correlation analyses with significant interaction with Aβ status were mark with an asterisk (*). Age and sex were used as covariates. Intracranial volume and follow‐up time were included as an additional covariate for cross‐sectional volume measurements and longitudinal measurements respectively. Years of education was included as an additional covariate for analyses of cognition. Scatter plots are shown in Figure S4 in supporting information. See Figure 1 for biomarker abbreviations. Structural MRI, cognition, and tau PET measures are color‐coded in green, red, and purple, respectively, for easier interpretation in all tables and figures.

Abbreviations: A+/A–, amyloid beta positive/negative; Aβ, amyloid beta; CDRSB, Clinical Dementia Rating Sum of Boxes; CU, cognitively unimpaired; DEL, memory delayed recall; MMSE, Mini‐Mental State Examination; MRI, magnetic resonance imaging; MTL, medial temporal lobe; PET, positron emission tomography; PHCVol, parahippocampal cortex volume.

While the separate model approach allows for explicitly testing our conceptual hypothesis within Aβ subgroups—those on the AD continuum (A+) and those not (A–)—it does not allow us to determine whether Aβ status significantly modifies the relationships among biomarkers. To address this, we performed linear regression for the entire CU cohort using the lm function in R for correlation analysis of each pair of biomarkers mentioned above. The two targeted biomarkers were set as dependent and independent variables, with Aβ included as an additional independent variable. An interaction term between Aβ status and the independent biomarker was incorporated to test for interaction effects. Covariates were selected in the same manner as previously described. Significant interactions are reported in Table 3.

**FIGURE 3 alz14492-fig-0003:**
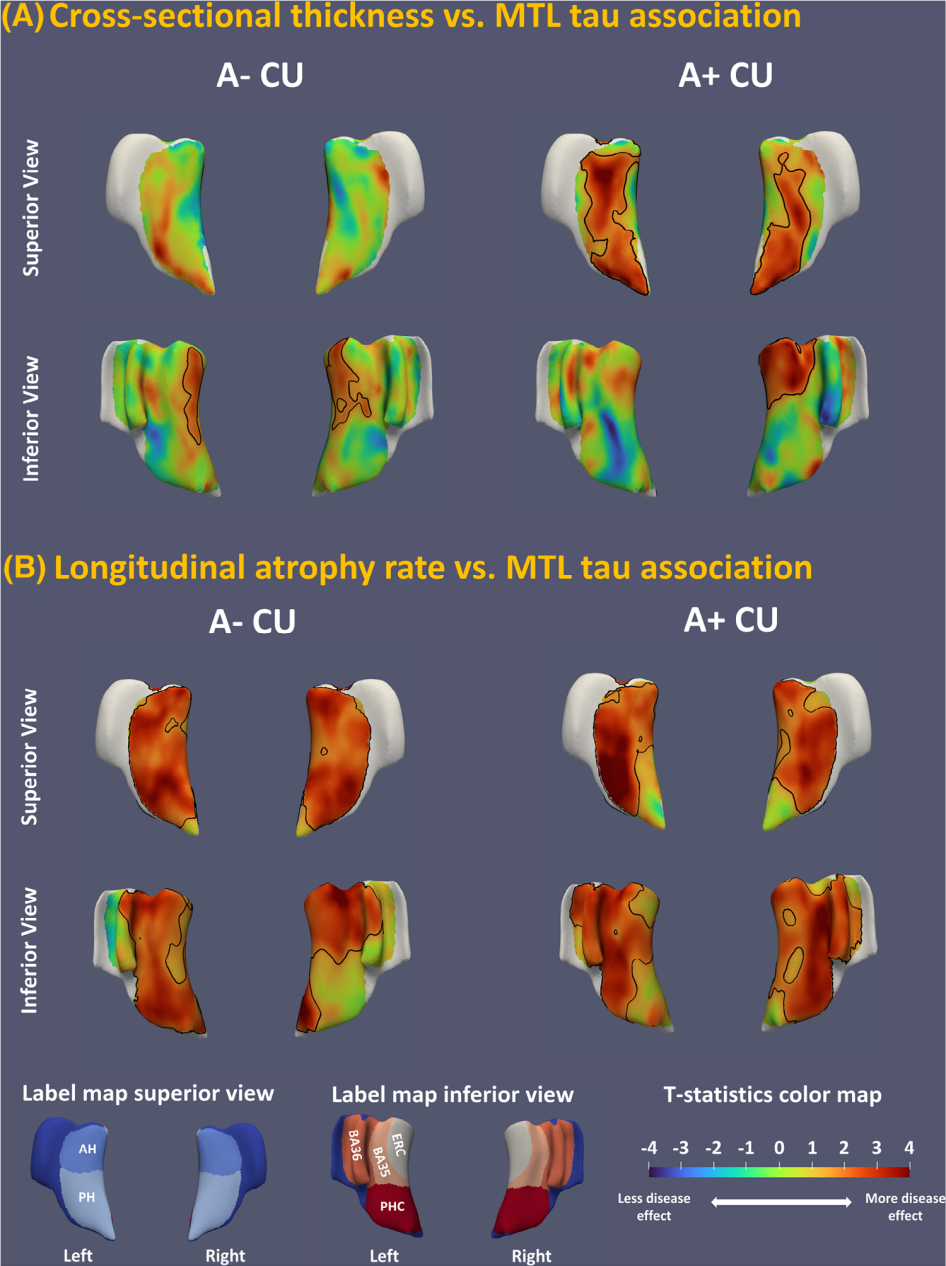
Two views of the statistical maps of association between tau tracer uptake in ERC and BA35 and cross‐sectional thickness (A) or longitudinal atrophy rate (B) structural measures. Analyses were done separately in Aβ positive/negative (A+/A–) CU subgroups. Each statistical map plots the *t* statistics associated with the null hypothesis that there is no correlation between structural measurement (either cross‐sectional thickness or longitudinal atrophy rate) and MTL tau tracer uptake. Clusters highlighted by black contours are statistically significant at *p* < 0.05 after correction for multiple comparisons using cluster‐level FWER permutation test approach with 1000 permutations and cluster threshold uncorrected *p* < 0.05. A+/A–, amyloid beta positive/negative; Aβ, amyloid beta; AH, anterior hippocampus; BA35/36, Brodmann area 35/36; CU, cognitively unimpaired; ERC, entorhinal cortex; FWER, family‐wise error rate; MTL, medial temporal lobe; PH, posterior hippocampus; PHC, parahippocampal cortex; T+/T–, tau positive/negative

In addition to ROI analyses, regional analyses were performed between tau burden and MRI‐based structural measures. GLMs were performed at each point on the MTL surface with tau PET measure as the independent variable, cross‐sectional thickness or longitudinal atrophy rate as the dependent variable, and age and sex as covariates. Regional analyses were done separately for each hemisphere with the corresponding tau burden measure in the MTL. In both analyses, ICV and follow‐up time were included as additional covariates in cross‐sectional volume and longitudinal measurements, respectively. Analyses were performed separately in A+ and A– CU subgroups with results reported in Figure 3.

We note that the tau‐anchored MRI scan was used as the baseline scan in analyses involving tau measures. Therefore, some MRI scans used to extract structural measurements were different from those that were used to perform group comparisons (see section 2.5.1). The maximum time difference between tau PET and the anchored MRI scan was set to 1 year.

#### 2.5.3 Analysis of the predictive power of baseline biomarkers in disease progression

Stepwise linear mixed effect modeling (lme4 package in R) was performed to identify the optimal model with a subset of baseline structural MRI, tau PET, and cognitive biomarkers in predicting future disease progression. DEL was used to estimate longitudinal cognitive decline, given the focus on the MTL in this study. CDRSB was used as a measure of longitudinal decline in cognition and function, given its clinical significance. Longitudinal BA35 volume change served as the proxy for progressive neurodegeneration as it showed the strongest effect between A+ and A– CU among all MTL subregions (Table 1) and was found to be a highly sensitive region to preclinical AD reported in our prior work.^8^, ^38^ Analyses were performed separately in the A+ and A– subgroups of CU. In total, six models were generated: two models (A+ CU and A– CU) for each of the three longitudinal biomarkers. Results are reported in Table 4.

**TABLE 4 alz14492-tbl-0004:** Results of the stepwise linear mixed effect model analyses in the A+ and A– subgroups of CU older adults for longitudinal atrophy (longitudinal BA35Vol) and cognitive decline (longitudinal DEL, longitudinal CDRSB) measurements.

Dependent variable	Group	Model statistics	Baseline measurements that are included in the model	
Longitudinal BA35Vol	A– CU	*N* = 226 AIC = 326.2 *R* ^2^ = 0.99 AUC = 0.72	MTLTau	*β* = –0.02, *p* = 3.4 × 10^−5^
	A+ CU	*N* = 116 AIC = 159.8 *R* ^2^ = 0.99 AUC = 0.82	MTLTau MMSE	*β* = –0.02, *p* = 1.5 × 10^−4^ *β* = 0.01, *p* = 0.022
Longitudinal DEL	A– CU	*N* = 281 AIC = 1906.9 *R* ^2^ = 0.66 AUC = 0.59	MTLTau	*β* = –0.03, *p* = 0.028
	A+ CU	*N* = 141 AIC = 1083.1 *R* ^2^ = 0.60 AUC = 0.72	MMSE MTLTau	*β* = 0.07, *p* = 3.4 × 10^−3^ *β* = –0.05, *p* = 0.038
Longitudinal CDRSB	A– CU	*N* = 295 AIC = 1949.2 *R* ^2^ = 0.61 AUC = 0.64	MTLTau MMSE	β = 0.07, *p* = 1.2 × 10^−3^ β = –0.04, *p* = 0.049
	A+ CU	*N* = 144 AIC = 977.1 *R* ^2^ = 0.70 AUC = 0.73	MTLTau DEL MMSE	*β* = 0.11, *p* = 3.9 × 10^−4^ *β* = –0.08, *p* = 9.3 × 10^−3^ *β* = –0.08, *p* = 0.013

*Note*: Variables fixed in the model: age, sex, education, *APOE* ɛ4 status, and follow‐up time. Baseline cross‐sectional variables: MRI (green), tau PET (purple), and cognitive (red) biomarkers in Figure 1. Tau PET and cognitive biomarkers provide complementary information in the prediction. See Figure 1 for biomarker abbreviations. Structural MRI, cognition and tau PET measures were color‐coded in green, red and purple respectively for easier interpretation in all tables and figures.

Abbreviations: AIC, Akaike information criterion; *APOE*, apolipoprotein E; AUC, area under the curve; A+/A–, amyloid beta positive/negative; CDRSB, Clinical Dementia Rating Sum of Boxes; CU, cognitively unimpaired; DEL, memory delayed recall; MMSE, Mini‐Mental State Examination; MRI, magnetic resonance imaging; MTL, medial temporal lobe; PET, positron emission tomography.

To investigate whether selection of subregion other than BA35 significantly impacts the results, we performed additional analyses using longitudinal ERC and posterior hippocampal volume changes as dependent variables. These additional results are included in Table S1 in supporting information.

Specifically, the following steps were performed for each model fitting: (1) The baseline biomarkers were normalized by having their mean subtracted and then divided by the SD. (2) A base linear mixed effect model was established, incorporating fixed effects including age, sex, education, *APOE* ɛ4 status, and follow‐up time. Random effects included a random intercept and a random slope for time from baseline for each participant. (3) The model was then updated iteratively. In each iteration, each of the remaining baseline measurements was added to the model generated in the last iteration, along with an interaction term with time from baseline. After evaluating each of the remaining baseline measurements, one at a time, the baseline measurement that resulted in significant model improvement and the greatest increase of the Akaike information criterion (AIC) was permanently added to the model generated in the last iteration, yielding the current best model in each iteration. Because only the best baseline measure was selected from the remaining ones, there were no effects of the order of baseline measurements investigated in each iteration in this stepwise analysis. (4) The iterative process was halted if no remaining baseline measurements significantly improved the model. The current best model was then considered the final best model. A complete list of dependent variables, fixed effects, random effects, and independent variable candidates is summarized in Table S2 in supporting information.

**FIGURE 4 alz14492-fig-0004:**
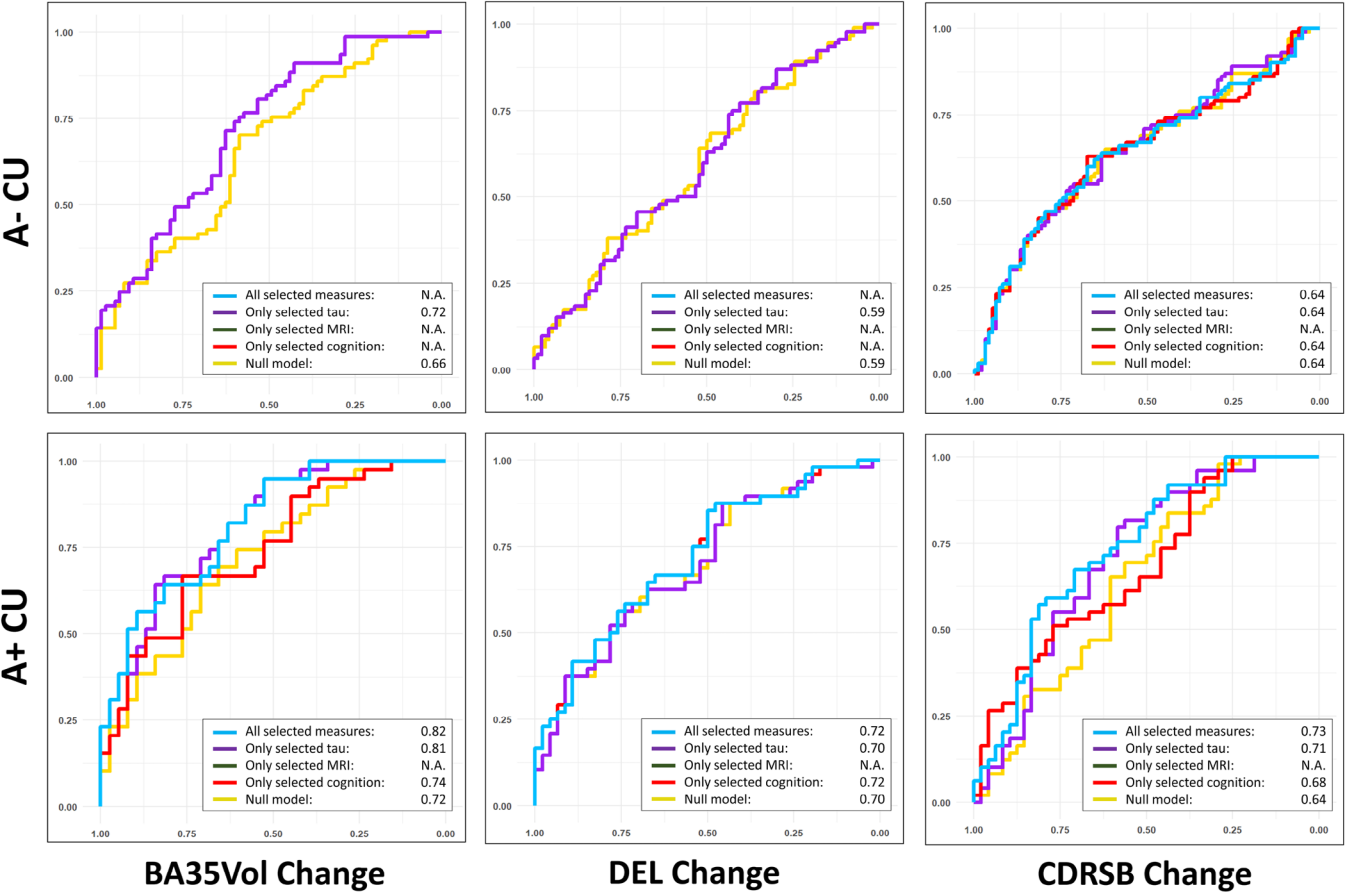
ROC analyses for discriminating fast versus slow progressors. Curves include models using none (yellow, null model with age, sex, education, *APOE* ɛ4 status, and follow‐up time), only tau‐based (purple), only MRI‐based (green), only cognition‐based (red), or all the (light blue) selected baseline cross‐sectional biomarkers in Table 4. Analyses were done in A– (first row) and A+ (second row) CU separately. Tau PET and cognitive biomarkers provided complementary information in the predictions. A+/A–, CU. See Figure 1 for biomarker abbreviations. Structural MRI, cognition and tau PET measures were color‐coded in green, red and purple respectively for easier interpretation in all tables and figures. A+/A–, amyloid beta positive/negative; *APOE*, apolipoprotein E; CU, cognitively unimpaired; PET, positron emission tomography; ROC, receiver operating characteristic

In addition, to investigate the power of baseline measurements in discriminating fast from slow progressors (defined by the first and last tercile) in each Aβ subgroup (A– CU and A+ CU), we computed a summary measurement for each Aβ subgroup by multiplying the coefficients of the final best model to the corresponding selected baseline biomarkers. Then, for each model, the receiver operating characteristic (ROC) analysis was performed with the summary measurement and the area under the curve (AUC) was reported. This analysis was performed separately for each of the six best final models for all three longitudinal measures and the two Aβ subgroups of CU, as well as for each of the four final best models in Table S2. For comparisons, each analysis was repeated with no baseline biomarker (denoted as the null model) or only one of the selected structural MRI, tau PET, or cognitive biomarkers in the model. Results are reported in Figure 4 and Figure S6 in supporting information.[Table alz14492-tbl-0003], [Fig alz14492-fig-0003]

## 3 RESULTS

Basic characteristics of the multisite dataset of CU individuals are summarized in Table 1. Out of the 3036 participants, 1558 had positive amyloid status (51.3%) and 1270 were amyloid negative (48.7%). A total of 208 participants (A4/HABS/ADNI/ABC: 0/1/88/119) did not have Aβ status available. A+ CU individuals were significantly older than A– CU (72.6 vs. 71.3 years old, *p* < 0.001, Cohen *d* = 0.23). The proportion of *APOE* ɛ4 carriers in A+ CU was significantly higher than that in A– CU (56.7% vs. 22.0%, *p* < 0.001). There was no significant difference in years of education, sex, race, ethnic groups, and baseline CDRSB performance between A+ and A– CU subgroups.

### 3.1 Biomarker analyses in A and T subgroups of CU individuals

We first performed biomarker analyses between Aβ subgroups. As shown in Table 1 and Figure S2, significant differences between A+ and A– CU were observed in cross‐sectional tau, cross‐sectional and longitudinal cognition, as well as cross‐sectional and longitudinal structural MRI measures. Among all the measurements, the cross‐sectional tau burden in the MTL demonstrated the strongest difference in absolute terms between A+ and A– CU (*p* < 0.001, Cohen *d* = 0.50). As for structural MRI measures, the effects of longitudinal atrophy were consistently stronger than cross‐sectional ones in all subregions (*p* < 0.05 for comparison of corresponding cross‐sectional and longitudinal Cohen *d* values of each subregion). Longitudinal BA35 volume change was the strongest (*p* < 0.001, Cohen *d* = 0.46) among longitudinal structural measures in absolute terms. The effect sizes of cognitive measures (Cohen *d* = 0.1–0.28) were smaller than that of cross‐sectional MTLTau (Cohen *d* = 0.50, *p* < 0.05 in Cohen *d* comparison) and longitudinal structural MRI measures (Cohen *d* = 0.32–0.46, in absolute terms).[Table alz14492-tbl-0004], [Fig alz14492-fig-0004]

When further divided into A and T subgroups (Table 2 and Figure S3), the effects between A+ and A– CU (Table 1) were mainly driven by A+T+ CU individuals, as this group had the biggest difference compared to A–T– CU. Significantly faster longitudinal atrophy rate in ERC and BA36 measured by structural MRI was observed even in participants with no evidence of Aβ pathology but who were tau PET positive (A–T+ CU) compared to A–T– CU, potentially indicating direct linkage between tau burden and brain structure in participants without AD.

For cross‐sectional and longitudinal structural MRI biomarkers, we additionally performed regional surface‐based analyses to investigate patterns of disease effects in the MTL, which are summarized in Figure 2. The average regional cross‐sectional thickness and longitudinal atrophy rate maps can be found in Figure S1 with the fastest longitudinal atrophy rate observed in ERC and BA35 in both A– and A+ CU. Overall, the results were consistent with ROI analyses. The group differences of longitudinal structural MRI biomarkers were stronger than cross‐sectional measures in absolute terms (Figure 2B vs. Figure 2A). Longitudinal atrophy in A+ CU was widespread in the MTL with the effect in ERC, BA35 being the strongest in terms of having higher *t* statistics (first column of Figure 2B). When further divided into A and T subgroups, we can observe significant differences using longitudinal MTL atrophy measures in all subgroup comparisons, including A–T– CU versus A+T– CU and A+T– CU versus A+T+ CU (the third and fourth columns in Figure 2B).

### 3.2 Correlation among tau, structure in the MTL subregions, and cognitive measures

Pearson partial correlation analyses (Table 3 and Figure S4) demonstrated significant associations between cross‐sectional tau burden in the MTL and both cross‐sectional (all subregions except PHC thickness with ρ from –0.11 to –0.26) and longitudinal MTL structural measures (all subregions with ρ from –0.28 to –0.39) in A+ CU, highlighted by the orange background in Table 3. Interestingly, significant correlations were observed in the A– CU subgroup [cross‐sectional PHVol (ρ = –0.15, *p* < 0.001), ERCThk (ρ = –0.13, *p* = 0.003), and BA36Thk (ρ = –0.13, *p* = 0.003) as well as longitudinal AHVolChg (ρ = –0.17, *p* = 0.006) and ERCVolChg (ρ = –0.24, *p* < 0.001)], that is, CU participants without evidence of Aβ pathology, which may reflect the effect of primary age‐related tauopathy (PART) that is common in A– individuals within the age range of the current dataset, low levels of Aβ pathology that do not meet the threshold of amyloid positivity, or non‐specific binding related to other pathologies,^39^ such as TDP‐43 (see Discussion section). However, only a few of these relationships with MTLTau were associated with a significant interaction with Aβ status; namely, AH in the cross‐sectional analysis and BA36 in the longitudinal analysis with MTLTau were significant in which there was a greater slope for A+ than A– individuals. While other interactions were not significant, there was a trend in structural longitudinal analyses for most associations with tau to have a higher slope in the A+ than A– CU groups. Regional analyses results (Figure 3) replicated the results in the ROI analysis, confirming the widespread effects in the MTL with hotspots in the posterior hippocampus, ERC, and BA35. Overall, stronger correlations were observed for longitudinal MTL structural measures compared to cross‐section structural ones.

Significant correlations between MTLTau and CDRSBChg were observed irrespective of Aβ status (A– CU: ρ = 0.15, *p* = 0.009; A+ CU: ρ = 0.30, *p* < 0.001). All other tau–cognition correlations were not significant. For cross‐sectional PHVol, the Pearson correlation coefficients were either very small or just reached significance. The strongest correlation was with longitudinal CDRSBChg in A+ CU individuals (ρ = 0.19, *p* = 0.002). Significant interactions were seen in correlations with cross‐sectional DEL as well as longitudinal change of MMSE and CDRSB, indicating significantly steeper slope in the A+ CU subgroup. A post hoc mediation analysis showed that the relationship between MTLTau and CDRSBChg in A+ CU was partially mediated by PHVol (Figure S5).

### 3.3 Predictive power of baseline tau, cognition, and structural biomarkers in A+/A– CU

We investigated the predictive value of baseline measures for evidence of disease progression assessed by either memory or a global measure (longitudinal DEL change and CDRSB change) and structural atrophy (longitudinal BA35 volume change), summarized in Table 4. All models were significant, indicating that combinations of baseline biomarkers were predictive of disease progression in CU adults. MTLTau measure was selected in all the models (6 out of 6 models) and cognitive biomarkers provided additional information in 4 out of 6 models. Structural measures derived from MRI did not provide additional information on top of tau PET and cognitive measures in predicting longitudinal BA35 volume, DEL, and CDRSB change (the 6 models in Table 4 and Figure 4). However, structural MRI measures (baseline PHC thickness and posterior hippocampal volume) were selected when predicting longitudinal posterior hippocampal volume change and thus may provide some complementary information to tau PET (Table S1 and Figure S6).

When discriminating fast versus slow progressors (defined by the first and last tercile), the combination of tau PET, MRI measures, and cognitive biomarkers achieved the best results compared to the models using a single modality and the base models, with the best, but modest, prediction in the A+ CU subgroup in which AUCs ranged from 0.72 to 0.82 (row 2 in Figure 4) and more weakly in A– CU (ranging from 0.59 to 0.72).

## 4 DISCUSSION

The current study investigated AD neuroimaging and cognitive biomarkers in 3036 CU older adults. The findings demonstrated that cross‐sectional MTL tau burden and longitudinal MRI‐based atrophy of the MTL subregions best differentiated participants with and without Aβ (Table 1, Figure 2). Individuals with both cerebral amyloid and MTL tau positivity (A+T+ CU) exhibited an increased rate of neurodegeneration and subtle cognitive decline (in CDRSB) compared to A–T– CU (Table 2). In addition, an increased rate of neurodegeneration in A+T+ CU was also observed compared to those with cerebral amyloid who did not meet the threshold of MTL tau PET positivity (A+T– CU, the fourth column in Figure 2). This supports the role of tau pathology in driving neurodegeneration with the presence of amyloid and that tau PET (^18^F‐Flortaucipir) is sensitive to a meaningful level of MTL tau that portends this neurodegeneration. These findings closely align with other work, including that of Ossenkoppele et al., who reported faster cognitive decline, including progression to MCI, in 1325 CU individuals with PET evidence of MTL tau.^6^ The current work demonstrates the neurodegenerative correlates of their findings in the MTL, although with a partially overlapping cohort (HABS was in both studies). Interestingly, significantly faster atrophy rate in ERC and BA35 was also observed in A– CU with tau positivity (A–T+ CU) compared to A–T– CU (Table 2, Figure 2), potentially reflecting PART. Further, the MTL tau PET measures displayed a significant correlation with longitudinal MTL atrophy and rate of CDRSB change regardless of Aβ status (Table 3a, Figure 3). A significant association between posterior hippocampal volume and rate of CDRSB change was observed in A+ CU (Table 3b). A post hoc mediation analysis indicated posterior hippocampal volume mediated the correlation between MTL tau and CDRSB decline in A+ CU. A combination of baseline tau PET, structural MRI, cognitive measures, demographic information, and *APOE* ɛ4 genotype was predictive of cognitive and structural progression in CU older adults (Table 4, Figure 4). These results replicated prior findings and provided evidence to resolve contradictions in prior studies, which will be elaborated upon below.

### 4.1 Differences of tau, structural MRI, and cognitive measures in A and T subgroups of CU

Comparing cross‐sectional biomarkers in separating A+ and A– CU individuals, only small differences were observed in PH volume and BA35 thickness, reaffirming the findings in the smaller studies by Xie et al.^8^ and Koenig et al.^10^ The lack of a strong cross‐sectional effect among structural imaging measures, even in regions with the earliest tau pathology, is not surprising in the preclinical phase, during which the variance due to developmental differences and other factors over the lifespan are more likely to modulate structure relative to the signal related to AD‐related neurodegeneration.

Alternatively, and consistent with our prior results,^8^ longitudinal atrophy measures of the MTL subregions were much more sensitive to preclinical AD in both ROI (Table 1) and regional (Figure 2) analyses compared to cross‐sectional ones (statistically significant). Longitudinal atrophy rate in BA35 was the strongest measure (Cohen *d* = 0.46) among MTL subregions in absolute terms, which is consonant with the fact that the transentorhinal cortex (largely overlapping with BA35) is the first cortical site of tau pathology.^9^ The effects of MTL longitudinal atrophy were mostly driven by A+T+ CU individuals (Table 2 and Figure 2), supporting the prognostic value of the amyloid/tau/neurodegeneration framework^40^ and more recent AD staging system in preclinical population (aaic.alz.org/diagnostic‐criteria.asp).^7^

Significant group effects were seen in both cross‐sectional (MMSE, DEL) and longitudinal (DEL change rate and CDRSB change rate) cognitive biomarkers (Table 1). However, their ability to discriminate Aβ CU subgroups was inferior to both cross‐sectional tau PET (statistically significant) and longitudinal structural MRI measures (in absolute terms). This is expected in a CU population and, again, supports biological staging of disease as providing additional information beyond cognitive measures.^41^

### 4.2 Correlations among tau PET, structural MRI, and cognitive biomarkers

Significant correlations were found between tau PET and cross‐sectional structural MRI and with prospective longitudinal atrophy rates in CU older adults (Table 3 and Figure 3). In most MTL subregions, tau structural correlations were stronger in A+ CU compared to A– CU, consistent with previous work;^11^, ^12^ however, only a subset of these reached statistical significance. Nonetheless, the associations were significant regardless of Aβ status, replicating findings in Maass et al.^12^ The pattern of most significant association with MTL tau PET, that is, longitudinal atrophy rate in BA35 and along the long axis of the hippocampus in both groups (Figure 3B), is consistent with the tau distribution described by Braak and Braak^9^ and other *post mortem* studies.^42^ This indicates AD‐like neurodegeneration in A– CU and suggests the possibility that it could be driven by PART, resembling findings in Wuestefeld et al.^43^, ^44^ While low levels of ^18^F‐Flortaucipir uptake have been related to non‐specific neurodegeneration, including semantic dementia associated with TDP‐43 pathology, the neurodegenerative pattern here seems more aligned with the distribution of tau pathology rather than other MTL processes, such as limbic‐predominant age‐related TDP‐43 encephalopathy (LATE),^45^, ^46^ which has been described to be associated with more severe atrophy in anterior hippocampal relative to AD‐related tau.^47^ However, a recent *post mortem* study by Josephs et al.^48^ reported that *ante mortem* ^18^F‐Flortaucipir tracer uptake was not associated with “definite” PART [defined as Thal or Consortium to Establish a Registry for Alzheimer's Disease (CERAD score of 0] but showed some sensitivity to “possible” PART (defined as Thal 1–2 or CERAD 1). Note that this study operationalized severity as Braak stage, but not continuous measure of tau load in the MTL, which may influence tracer uptake and related neurodegeneration. Nonetheless, as the threshold of amyloid PET is generally insensitive to the Thal < 3 or CERAD < 2, it is certainly possible that the cases driving the above correlation in the CU (A– CU) would fall in the possible PART category, which also overlaps with low level of AD neuropathologic change (ADNC) and reflects a boundary zone between AD and PART. Though we cannot pinpoint the degree to which varying levels of PART, non‐specific uptake, or some combination thereof is the driver of the relationship, the increased MTL tau tracer uptake in A– CU is not a completely benign state.

While structural MTL MRI measures generally had much weaker correlations with cognition than with tau PET, the results of this study also support the notion that tau's effect on cognition is mediated, at least in part, through neurodegeneration. While this could reflect a temporal lag between the development of neurofibrillary tangles and observable neurodegeneration, it also may reflect signal‐to‐noise differences of the measures in the preclinical stage of AD when structural changes may be overwhelmed by other sources of interindividual variability noted above. Regardless, posterior hippocampal volume still demonstrated a mediating effect between tau PET and cognition in Aβ‐positive CU subgroups. This echoes the causal linkage between tau and neurodegeneration reported by Bilgel et al.^49^ Given the relatively low explained variance in the mediation model by posterior hippocampal volume, it seems likely that tau pathology exerts its effect on cognition through other mechanisms than neurodegeneration, such as changes in synaptic function.

### 4.3 Tau burden in the MTL is the strongest predictor of disease progression in CU

Stepwise linear mixed‐effect analyses (Table 4, Figure 4, Table S1 and Figure S6) demonstrated the significant value of MTL tau burden in predicting both longitudinal structural atrophy and future cognitive decline given that it was selected in all the models regardless of Aβ status. Such a result is consistent with the group effects of demonstrated T+ cases having more extensive atrophy and, to a lesser extent, clinical progression than T– cases, as has also been reported in other work.^6^, ^15^ The current work directly compared the prognostic value of tau PET, structural MRI, and cognitive biomarkers and demonstrated the greater value of tau biomarkers in predicting disease progression in preclinical AD. Baseline cognitive and structural MRI measures provided additional information beyond tau PET for prediction, despite the limited performance in discriminating Aβ subgroups (Table 1). This is perhaps unsurprising, as poorer cognitive performance or faster structural changes may reflect early subtle changes of a more progressive stage of preclinical AD.

### 4.4 Limitations and future work

This study focused on neuroimaging and cognitive measures in CU older adults. Due to the limited availability of longitudinal tau PET, only a cross‐sectional tau measure was investigated. Biofluid biomarkers, particularly plasma, have demonstrated sensitivity to preclinical AD prediction^50^ and warrant future comparison with tau PET. Also, the cognitive measures were limited to those available across all four studies. Future investigations considering more sensitive cognitive measures, such as the Preclinical Alzheimer Cognitive Composite (PACC),^51^ could improve prediction in preclinical AD. Additionally, we focused on the MTL because of its early involvement in AD, but future studies should explore other AD‐associated brain regions. Moreover, the derived threshold to define tau positivity could be related to idiosyncratic aspects of the dataset, but our derived cutoff is similar to other studies examining MTL tau. Similarly, Aβ positivity was defined using cohort‐specific criteria, which introduced some inconsistency into the analysis. In addition, non‐White and non‐Hispanic populations are underrepresented, limiting the generalizability of our findings, highlighting the need for more diverse recruitment in future research.^52^

## 5 CONCLUSION

In conclusion, our multisite study of AD neuroimaging and cognitive biomarkers in 3036 CU individuals demonstrated the utility of multimodal biomarkers in characterizing preclinical AD and normal aging. The results demonstrated that MTL tau is a primary driver of neurodegeneration in this region and reflected that T+ individuals are more likely to display evidence of neurodegenerative change and clinical decline proximally to those without tau detected by PET. As such, these measures may identify individuals most likely to benefit from interventions in the near term, as well as a potential means to monitor outcomes, notwithstanding the paradoxical effects of current anti‐amyloid therapies on brain structure.^53^ Our study also highlights the importance of large, multisite cohorts in validating and generalizing findings of biomarker research.

## CONFLICT OF INTEREST STATEMENT

D. A. W. has served as a paid consultant to Eli Lilly, GE Healthcare, and Qynapse. He serves on DSMB for Functional Neuromodulation and GSK. He is a site investigator for a clinical trial sponsored by Biogen. S. R. D. received consultation fees from Rancho Biosciences and Nia Therapeutics. L. X. received personal consulting fees from Galileo CDS, Inc. L. X. has become an employee of Siemens Healthineers since May 2022 but the current study was conducted during his employment at the University of Pennsylvania. E. G. is a paid employee of Siemens Healthineers. The other authors have nothing to disclose. Author disclosures are available in the supporting information.

## CONSENT STATEMENT

Informed written consent was provided by all participants at each site of the ADNI, HABS, A4, and ABC studies.

## Supporting information



Supporting Information

Supporting Information

Supporting Information

## References

[1] Sims JR , Zimmer JA , Evans CD , et al. Donanemab in early symptomatic Alzheimer disease: the TRAILBLAZER‐ALZ 2 randomized clinical trial. JAMA. 2023;330:512‐527. doi:10.1001/JAMA.2023.13239 37459141 PMC10352931

[2] van Dyck CH , Swanson CJ , Aisen P , et al. Lecanemab in early Alzheimer's disease. N Engl J Med. 2023;388:142‐143. doi:10.1056/NEJMOA2212948 36449413

[3] Sperling RA , Donohue MC , Raman R , et al. Trial of solanezumab in preclinical Alzheimer's disease. N Engl J Med. 2023;389:1096‐1107. doi:10.1056/NEJMOA2305032/SUPPL_FILE/NEJMOA2305032_DATA-SHARING.PDF 37458272 PMC10559996

[4] Falcon C , Tucholka A , Monté‐Rubio GC , et al. Longitudinal structural cerebral changes related to core CSF biomarkers in preclinical Alzheimer's disease: a study of two independent datasets. Neuroimage Clin. 2018;19:190‐201. doi:10.1016/J.NICL.2018.04.016 30023169 PMC6050455

[5] Pegueroles J , Vilaplana E , Montal V , et al. Longitudinal brain structural changes in preclinical Alzheimer's disease. Alzheimers Dement. 2017;13:499‐509. doi:10.1016/J.JALZ.2016.08.010 27693189

[6] Ossenkoppele R , Pichet Binette A , Groot C , et al. Amyloid and tau PET‐positive cognitively unimpaired individuals are at high risk for future cognitive decline. Nat Med. 2022;28:2381‐2387. doi:10.1038/s41591-022-02049-x 36357681 PMC9671808

[7] Jack CR , Andrews JS , Beach TG , et al. Revised criteria for diagnosis and staging of Alzheimer's disease: Alzheimer's association workgroup. Alzheimers Dement. 2024;20(8):5143‐5169. doi:10.1002/ALZ.13859 38934362 PMC11350039

[8] Xie L , Wisse LEM , Das SR , et al. Longitudinal atrophy in early Braak regions in preclinical Alzheimer's disease. Hum Brain Mapp. 2020;41(16):4704‐4717. doi:10.1002/hbm.25151 32845545 PMC7555086

[9] Braak H , Braak E . Staging of Alzheimer's disease‐related neurofibrillary changes. Neurobiol Aging. 1995;16:271‐278. 7566337 10.1016/0197-4580(95)00021-6

[10] Koenig LN , LaMontagne P , Glasser MF , et al. Regional age‐related atrophy after screening for preclinical alzheimer disease. Neurobiol Aging. 2022;109:43‐51. doi:10.1016/J.NEUROBIOLAGING.2021.09.010 34655980 PMC9009406

[11] Berron D , Vogel JW , Insel PS , et al. Early stages of tau pathology and its associations with functional connectivity, atrophy and memory. Brain. 2021;144(9):2771‐2783. doi:10.1093/brain/awab114 33725124 PMC8557349

[12] Maass A , Lockhart SN , Harrison TM , et al. Entorhinal tau pathology, episodic memory decline and neurodegeneration in aging. J Neurosci. 2017;38:2028‐2017. doi:10.1523/JNEUROSCI.2028-17.2017 PMC5777108 29192126

[13] Simon SS , Varangis E , Lee S , et al. In vivo tau is associated with change in memory and processing speed, but not reasoning, in cognitively unimpaired older adults. Neurobiol Aging. 2024;133:28‐38. doi:10.1016/J.NEUROBIOLAGING.2023.10.001 38376885 PMC10879688

[14] Rani N , Alm KH , Corona‐Long CA , et al. Tau PET burden in Brodmann areas 35 and 36 is associated with individual differences in cognition in non‐demented older adults. Front Aging Neurosci. 2023;15:1272946. doi:10.3389/FNAGI.2023.1272946/FULL 38161595 PMC10757623

[15] Fujishima M , Kawasaki Y , Mitsuhashi T , Matsuda H . Impact of amyloid and tau positivity on longitudinal brain atrophy in cognitively normal individuals. Alzheimers Res Ther. 2024;16:1‐16. doi:10.1186/S13195-024-01450-7/TABLES/3 38600602 PMC11005141

[16] Insel PS , Donohue MC , Sperling R , Hansson O , Mattsson‐Carlgren N . The A4 study: β‐amyloid and cognition in 4432 cognitively unimpaired adults. Ann Clin Transl Neurol. 2020;7:776‐785. doi:10.1002/ACN3.51048 32315118 PMC7261742

[17] Dagley A , LaPoint M , Huijbers W , et al. Harvard aging brain study: dataset and accessibility. Neuroimage. 2017;144:255‐258. doi:10.1016/J.NEUROIMAGE.2015.03.069 25843019 PMC4592689

[18] Wechsler D . Wechsler Memory Scale‐Revised Manual. Springer; 1987.

[19] Royse SK , Minhas DS , Lopresti BJ , et al. Validation of amyloid PET positivity thresholds in centiloids: a multisite PET study approach. Alzheimers Res Ther. 2021;13:1‐10. doi:10.1186/S13195-021-00836-1/FIGURES/4 33971965 PMC8111744

[20] Collij LE , Bischof GN , Altomare D , et al. Quantification supports amyloid PET visual assessment of challenging cases: results from the AMYPAD diagnostic and patient management study. J Nucl Med. 2024. doi:10.2967/JNUMED.124.268119 PMC11705786 39542700

[21] Joie RL , Mundada NS , Blazhenets G , et al. Quantitative amyloid‐PET in real‐world practice: lessons from the imaging dementia—evidence for amyloid scanning (IDEAS) study. Alzheimers Dement. 2023;19:e082874. doi:10.1002/ALZ.082874

[22] Xie L , Wisse LEM , Das SR , et al. Accounting for the confound of meninges in segmenting entorhinal and perirhinal cortices in T1‐weighted MRI. Med Image Comput Comput Assist Interv. 2016;9901:564‐571. doi:10.1007/978-3-319-46723-8_65 28752156 PMC5526195

[23] Xie L , Wisse LEM , Pluta J , et al. Automated segmentation of medial temporal lobe subregions on in vivo T1‐weighted MRI in early stages of Alzheimer's disease. Hum Brain Mapp. 2019;40:3431‐3451. doi:10.1002/hbm.24607 31034738 PMC6697377

[24] Xie L , Pluta JB , Das SR , et al. Multi‐template analysis of human perirhinal cortex in brain MRI: explicitly accounting for anatomical variability. Neuroimage. 2017;144:183‐202. doi:10.1016/j.neuroimage.2016.09.070 27702610 PMC5183532

[25] Yushkevich PA , Xie L , Wisse LE , et al. Mapping medial temporal lobe longitudinal change in preclinical Alzheimer's disease. Alzheimers Dement. 2023;19:e081898. doi:10.1002/ALZ.081898

[26] Das SR , Avants BB , Pluta J , et al. Measuring longitudinal change in the hippocampal formation from in vivo high‐resolution T2‐weighted MRI. Neuroimage. 2012;60:1266‐1279. doi:10.1016/j.neuroimage.2012.01.098 22306801 PMC3667607

[27] Avants BB , Epstein CL , Grossman M , Gee JC . Symmetric diffeomorphic image registration with cross‐correlation: evaluating automated labeling of elderly and neurodegenerative brain. Med Image Anal. 2008;12:26‐41. doi:10.1016/j.media.2007.06.004 17659998 PMC2276735

[28] Yushkevich PA , Pluta J , Wang H , Wisse LEM , Das S , Wolk D . Fast automatic segmentation of hippocampal subfields and medial temporal lobe subregions in 3 tesla and 7 tesla T2‐weighted MRI. Alzheimers Dement. 2016;12:P126‐P127. doi:10.1016/j.jalz.2016.06.205

[29] Reynolds D . Gaussian mixture models. Encyclopedia Of Biometrics. Springer; 2009:659‐663. doi:10.1007/978-0-387-73003-5_196

[30] Strikwerda‐Brown C , Hobbs DA , Gonneaud J , et al. Association of elevated amyloid and tau positron emission tomography signal with near‐term development of Alzheimer disease symptoms in older adults without cognitive impairment. JAMA Neurol. 2022;79:975‐985. doi:10.1001/JAMANEUROL.2022.2379 35907254 PMC9339146

[31] Franzmeier N , Dewenter A , Frontzkowski L , et al. Patient‐centered connectivity‐based prediction of tau pathology spread in Alzheimer's disease. Sci Adv. 2020;6:eabd1327. doi:10.1126/SCIADV.ABD1327/SUPPL_FILE/ABD1327_SM.PDF 33246962 PMC7695466

[32] Vogel JW , Iturria‐Medina Y , Strandberg OT , et al. Spread of pathological tau proteins through communicating neurons in human Alzheimer's disease. Nat Commun. 2020;11(1):2612. doi:10.1038/S41467-020-15701-2 32457389 PMC7251068

[33] Josephs KA , Weigand SD , Whitwell JL . Characterizing amyloid‐positive individuals with normal tau PET levels after 5 years: an ADNI study. Neurology. 2022;98:E2282‐E2292. doi:10.1212/WNL.0000000000200287 35314506 PMC9162162

[34] Weintraub S , Besser L , Dodge HH , et al. Version 3 of the Alzheimer disease centers’ neuropsychological test battery in the uniform data set (UDS). Alzheimer Dis Assoc Disord. 2018;32:10‐17. doi:10.1097/WAD.0000000000000223 29240561 PMC5821520

[35] Fortin J‐P , Cullen N , Sheline YI , et al. Harmonization of cortical thickness measurements across scanners and sites. Neuroimage. 2018;167:104. doi:10.1016/J.NEUROIMAGE.2017.11.024 29155184 PMC5845848

[36] Nichols T , Hayasaka S . Controlling the familywise error rate in functional neuroimaging: a comparative review. Stat Methods Med Res. 2003;12:419‐446. 14599004 10.1191/0962280203sm341ra

[37] Jack CR , Bennett DA , Blennow K , et al. NIA‐AA research framework: toward a biological definition of Alzheimer's disease. Alzheimers Dement. 2018;14:535‐562. doi:10.1016/j.jalz.2018.02.018 29653606 PMC5958625

[38] Wolk DA , Das SR , Mueller SG , Weiner MW , Yushkevich PA . Alzheimer's disease neuroimaging initiative. Medial temporal lobe subregional morphometry using high resolution MRI in Alzheimer's disease. Neurobiol Aging. 2017;49:204‐213. doi:10.1016/j.neurobiolaging.2016.09.011 27836336 PMC5154888

[39] Lu M , Collins EC , Devous MD , et al. A review of the flortaucipir literature for positron emission tomography imaging of tau neurofibrillary tangles. Brain Commun. 2023;6:fcad305. doi:10.1093/BRAINCOMMS/FCAD305 38187878 PMC10768888

[40] Van Der Flier WM , Scheltens P . The ATN framework—moving preclinical Alzheimer disease to clinical relevance. JAMA Neurol. 2022;79:968‐970. doi:10.1001/JAMANEUROL.2022.2967 36214803

[41] Jack CR , Knopman DS , Jagust WJ , et al. Hypothetical model of dynamic biomarkers of the Alzheimer's pathological cascade. Lancet Neurol. 2010;9:119‐128. doi:10.1016/S1474-4422(09)70299-6 20083042 PMC2819840

[42] Yushkevich PA , Muñoz López M , Iñiguez De Onzoño Martin MM , et al. Three‐dimensional mapping of neurofibrillary tangle burden in the human medial temporal lobe. Brain. 2021;144:2784‐2797. doi:10.1093/BRAIN/AWAB262 34259858 PMC8783607

[43] Wuestefeld A , Pichet Binette A , Berron D , et al. Age‐related and amyloid‐beta‐independent tau deposition and its downstream effects. Brain. 2023;146:3192‐3205. doi:10.1093/BRAIN/AWAD135 37082959 PMC10393402

[44] Wisse LE , Xie L , Das SR , et al. Tau pathology mediates age effects on medial temporal lobe structure. Neurobiol Aging. 2022;109:135‐144. doi:10.1016/J.NEUROBIOLAGING.2021.09.017 34740075 PMC8800343

[45] Nelson PT , Schneider JA , Jicha GA , Duong MT , Wolk DA . When Alzheimer's is LATE: why does it matter?. Ann Neurol. 2023;94:211‐222. doi:10.1002/ANA.26711 37245084 PMC10516307

[46] Nelson PT , Dickson DW , Trojanowski JQ , et al. Limbic‐predominant age‐related TDP‐43 encephalopathy (LATE): consensus working group report. Brain. 2019;142:1503‐1527. doi:10.1093/BRAIN/AWZ099 31039256 PMC6536849

[47] de Flores R , Wisse LEM , Das SR , et al. Contribution of mixed pathology to medial temporal lobe atrophy in Alzheimer's disease. Alzheimers Dement. 2020;16:843‐852. doi:10.1002/ALZ.12079 32323446 PMC7715004

[48] Josephs KA , Tosakulwong N , Weigand SD , et al. Flortaucipir PET uncovers relationships between tau and amyloid‐β in primary age‐related tauopathy and Alzheimer's disease. Sci Transl Med. 2024;16:eado8076. doi:10.1126/SCITRANSLMED.ADO8076/SUPPL_FILE/SCITRANSLMED.ADO8076_MDAR_REPRODUCIBILITY_CHECKLIST.PDF 39047115 PMC11423951

[49] Bilgel M , Wong DF , Moghekar AR , Ferrucci L , Resnick SM . Causal links among amyloid, tau, and neurodegeneration. Brain Commun. 2022;4:fcac193. doi:10.1093/BRAINCOMMS/FCAC193 35938073 PMC9345312

[50] Aschenbrenner AJ , Li Y , Henson RL , et al. Comparison of plasma and CSF biomarkers in predicting cognitive decline. Ann Clin Transl Neurol. 2022;9:1739‐1751. doi:10.1002/ACN3.51670 36183195 PMC9639639

[51] Donohue MC , Sperling RA , Salmon DP , et al. The preclinical Alzheimer cognitive composite: measuring amyloid‐related decline. JAMA Neurol. 2014;71:961‐970. doi:10.1001/jamaneurol.2014.803 24886908 PMC4439182

[52] Rentería MA , Mobley TM , Evangelista ND , et al. Representativeness of samples enrolled in Alzheimer's disease research centers. Alzheimers Dement. 2023;15(2):e12450. doi:10.1002/DAD2.12450 PMC10242202 37287650

[53] Alves F , Kalinowski P , Ayton S . Accelerated brain volume loss caused by anti–β‐amyloid drugs a systematic review and meta‐analysis. Neurology. 2023;100:E2114‐E2124. doi:10.1212/WNL.0000000000207156/ASSET/045A36FE-9341-4278-B2E9-2A0174766CBC/ASSETS/IMAGES/LARGE/10TTU1.JPG 36973044 PMC10186239

