# Supplementary material for: A multi‐cohort study of longitudinal and cross‐sectional Alzheimer's disease biomarkers in cognitively unimpaired older adults
Source: Alzheimers Dement. 2025 Jan 27;21(2):e14492. doi: 10.1002/alz.14492 (PMC11848397; doi:10.1002/alz.14492)
Supplement: Supplementary file 1 — Supporting Information [file ALZ-21-e14492-s003.docx]

**Appendix**

**A1. Collaborators**

A complete listing of ADNI, A4 and HABS investigators can be found at in the following links:

- ADNI: http://adni.loni.usc.edu/wp-content/uploads/how_to_apply/ADNI_Acknowledgement_List.pdf
- A4: https://www.actcinfo.org/wp-content/uploads/2023/10/A4-LEARN-Study-Team-List-Longitudinal-as-of-May-2023-Journal-Version-1.pdf
- HABS: https://habs.mgh.harvard.edu/our-team/ respectively.
