# Supplementary material for: A multi‐cohort study of longitudinal and cross‐sectional Alzheimer's disease biomarkers in cognitively unimpaired older adults
Source: Alzheimers Dement. 2025 Jan 27;21(2):e14492. doi: 10.1002/alz.14492 (PMC11848397; doi:10.1002/alz.14492)
Supplement: Supplementary file 2 — Supporting Information [file ALZ-21-e14492-s002.docx]

**Supplementary material**

**S1. Alzheimer’s Disease Neuroimaging Initiative (ADNI) study**

Data used in the preparation of this article were obtained from the ADNI database (adni.loni.usc.edu). The ADNI was launched in 2003 as a public-private partnership, led by Principal Investigator Michael W. Weiner, MD. The primary goal of ADNI has been to test whether serial magnetic resonance imaging (MRI), positron emission tomography (PET), other biological markers, and clinical and neuropsychological assessment can be combined to measure the progression of mild cognitive impairment and early Alzheimer’s disease. For up-to-date information, see www.adni-info.org.

**S2. CRASHS Pipeline for T1-weighted MRI**

Cortical reconstruction for automatic segmentation of hippocampal subfields (CRASHS) is a software pipeline that applies surface-based cortical modeling and registration techniques, ubiquitous in whole-brain morphometry (Fischl 2012; Han et al. 2004; Huntenburg, Steele, and Bazin 2018), to the segmentations of medial temporal lobe (MTL) subregions generated by ASHS-T1. CRASHS uses triangle meshes to represent the surface between the gray matter in the MTL and most of the hippocampus and the adjacent white matter (gray/white surface); the surface between MTL gray matter and cerebrospinal fluid (pial surface); and the surface located roughly halfway between these surfaces (average or mid-surface). Deformable diffeomorphic surface registration is used to map inflated MTL mid-surfaces from individual participants to a population template. This section describes the individual steps in the CRASHS pipeline.

**Step 1. White Matter Segmentation.** The ASHS-T1 atlas set did not include a label for the white matter. Rather than modify the ASHS-T1 atlas set and rerun the ASHS-T1 segmentation, we added the white matter label to the existing ASHS-T1 segmentations as an additional post-processing step. The MTL white matter was labeled semi-automatically using ITK-SNAP in the ASHS-T1 atlas set in the space of the T1-weighted MRI scans because CRASHS was initially developed for modeling MTL cortex in T1-weighted MRI (Yushkevich et al. 2023). The nnU-Net pipeline (Isensee et al. 2021) was trained to perform white matter segmentation on image region of interest (ROI) centered on the ASHS-T1 segmentation of the MTL. For a given T1-weighted MRI scan with its corresponding ASHS-T1 segmentation, we extract the left and right MTL ROIs from the T1-weighted MRI and apply the trained nnU-Net to obtain the white matter segmentation.

**Step 2. White Matter Postprocessing.** The white matter label obtained from the nnU-Net is adjacent to the gray matter of the MTL cortex, but due to different processing streams (ASHS-T1 gray matter; nnU-Net for white matter), gaps remain between the two sets of labels. Simple postprocessing using mathematical morphology algorithms is performed to fill these gaps.

**Step 3. Surface Modeling and Inflation with NighRes.** The postprocessing steps outlined above culminate with the generation of probability maps for the MTL gray matter, including all ASHS-T1 gray matter segmentations; and the postprocessed MTL white matter. These probability maps are provided as inputs to the NighRes package (Huntenburg, Steele, and Bazin 2018), which includes algorithms for surface-based representation and inflation of gray/white, pial, and mid-surfaces of the cerebral cortex. First, the white matter segmentation undergoes topological correction to enforce spherical topology using a topology-constrained fast marching method (Bazin and Pham 2007). Next, the CRUISE algorithm (Han et al. 2004) is used to generate a level set representation of the gray/white surface and to deform this surface via a topology-preserving level set method through the gray matter, yielding a level set representation of the pial surface. The pial surface representation generated by CRUISE explicitly accounts for existence of sulcal fundi, even if those are not labeled in the input gray matter segmentation (Han et al. 2004; Huntenburg, Steele, and Bazin 2018). The level set representation of the geometric average of the gray/white and pial surfaces is also generated by CRUISE (referred to as “mid-surface” here). Surface inflation (Tosun et al. 2004) is applied to the mid-surface to unfold it and simplify its shape, facilitating visualization and registration.

**Step 4. Groupwise Registration to a Population Template.** To construct a template for groupwise registration of inflated MTL surfaces, we apply computational anatomy techniques based on large deformation diffeomorphic metric mapping (LDDMM) (Arguillere et al. 2015; Miller, Trouve, and Younes 2006). We applied steps 1-3 above to a set of 48 ASHS-T1 segmentations from ADNI (right and left, right flipped onto the left side) for template construction. Each MTL was represented by its inflated mid-surface, represented as a triangular mesh. Each triangle in the inflated average mesh was assigned a probability of belonging to each of the seven anatomical labels (six gray matter labels: AH, PH, ERC, BA35, BA36, PHC and the white matter label) based on intersecting the uninflated mid-surface mesh with the ASHS-T1 segmentation. Note that the white matter segmentation boundary consists of portion adjacent to the gray matter segmentation, as well as a portion not adjacent to any gray matter voxel. During CRUISE processing, this portion of the boundary does not propagate into gray matter and the pial surface and mid-surface outputs of CRUISE are the same as the gray/white surface output. This portion of the mid-surface is distant from any gray matter ASHS-T1 labels and is labeled white matter. It is part of the inflation and registration but is ignored during analysis of cortical thickness.

Linear and deformable registration between inflated labeled mid-surfaces used the varifold similarity metric (Charon and Trouvé 2013) to measure geometric alignment between two meshes. The varifold metric captures both surface-to-surface distance between triangular meshes and agreement in triangle orientation. In our implementation, we adjust the formulation varifold metric to additionally account for anatomical label similarity. Specifically, during the computation of the varifold inner product between two meshes, the contribution of each pair of triangles is weighted by the dot product of their label probability vectors, so that agreement between similarly labeled triangles is given a greater weight.

Groupwise registration proceeds as follows. First, we use the linear transformation between each ASHS-T1 segmentation and the ASHS-T1 volumetric brain template to roughly align all inflated surfaces. Then, we perform similarity registration between all pairs of meshes using the varifold metric, find the mesh (center mesh) for which the sum of the varifold metric after registration to other meshes is smallest, and designate this mesh as the template. We apply the similarity transform matching to align each mesh to the template mesh. This results in a global alignment of pose and size between the input meshes.

The template then undergoes deformable registration towards the input shapes. During each stage, geodesic shooting (Miller, Trouve, and Younes 2006; Arguillere et al. 2015) with parameters $p_{1},\ldots,p_{n}$ applied to the deformed template, and for each aligned inflated input mid-surface $j$, the varifold metric is computed with the template that is deformed again using momenta $p_{j}$. The sum of the varifold metric across all input shapes forms the data term of the loss function that is minimized with respect to the parameters $p_{1},\ldots,p_{n}$. An additional loss term penalizes the total deformation energy (Hamiltonian of the geodesic shooting system). Gradient descent is used to update the parameters $p_{1},\ldots,p_{n}$. After each epoch of optimization, the parameters $p_{1},\ldots,p_{n}$ are averaged and the average parameters are applied to the template, propelling its shape towards the average of the input shape. Deformable registration is implemented in the Kernel Operations (KeOps) library (Charlier et al. 2021) that allows fast gradient computation for the geodesic shooting algorithm on the GPU with limited memory.

When processing the multi-site dataset, similar registration is performed between each inflated average surface extracted from the ASHS-T1 segmentation and the template. First, similarity registration is performed using the varifold metric. Then, geodesic shooting is performed between the template and the target surface, using a loss that combines the varifold metric, the deformation energy term, and the Jacobian term in the same way as during template construction. Finally, each point on the deformed template is matched to its closest point on the inflated average surface. This matching accounts for residual misregistration between the template and the target surface and ensures that each vertex in the template maps to a surface point on the inflated input mesh, rather than in the ambient space. Using this mapping and barycentric coordinate interpolation, the template can be projected back to the uninflated mid-surface of the input ASHS-T1 segmentation. This makes it possible for any data defined in the space of the input T1-weighted MRI, such as cortical thickness, to be mapped to the template.

**Step 5. Gray Matter Thickness Computation.** The gray matter thickness is computed from the ASHS-T1 segmentation. The gray matter labels are combined and the Voronoi diagram of this segmentation is computed. Each tetrahedron in the Voronoi diagram is associated with an inscribed sphere in the segmentation and the radius of this sphere is a local representation of thickness. By computing the intersection between the template mapped into the space of the uninflated average surface and the Voronoi tetrahedra, each point on the template can be associated with a radius (thickness value).


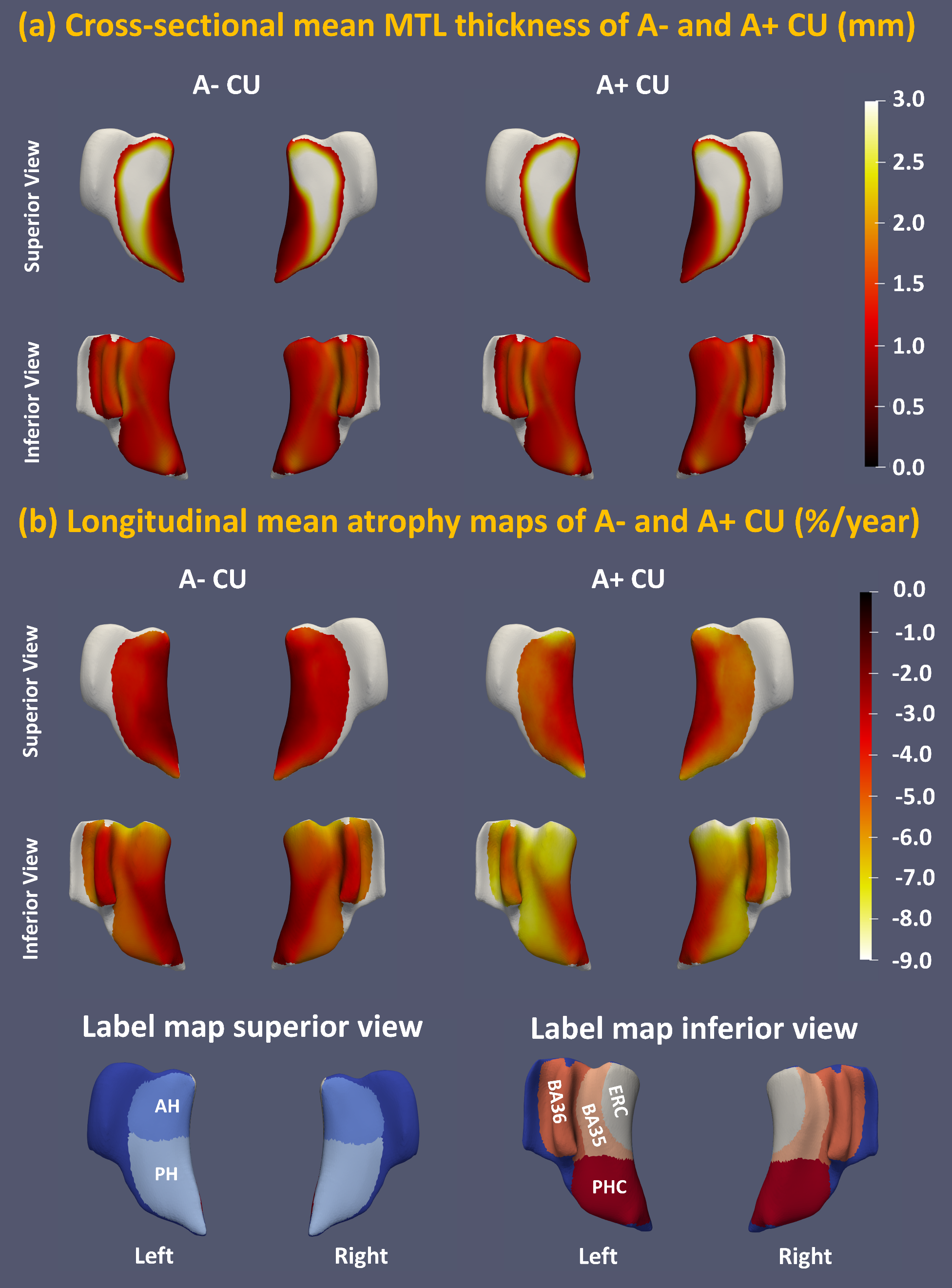


**Supplementary Figure S1.** Mean thickness (a) and atrophy rate (b) maps in the medial temporal lobe (MTL) of β-amyloid negative (A-) and positive (A+) subgroups of cognitively unimpaired (CU) older adults. AH/PH = anterior/posterior hippocampus; ERC = entorhinal cortex; BA35/36 = Brodmann areas 35/36; PHC = parahippocampal cortex.





**Supplementary Figure S2.** Bar plots comparing cross-sectional (a) and longitudinal (b) biomarkers of β-amyloid (A+/A-) subgroups of cognitively unimpaired (CU) individuals. See Figure 1 for biomarker abbreviations. Structural MRI, cognition and tau PET measures were color-coded in green, red and purple respectively for easier interpretation in all tables and figures.





**Supplementary Figure S3.** Bar plots comparing cross-sectional (a) and longitudinal (b) biomarkers of β-amyloid (A+/A-) and tau (T-/T+) subgroups of cognitively unimpaired (CU) individuals. See Figure 1 for biomarker abbreviations. Structural MRI, cognition and tau PET measures were color-coded in green, red and purple respectively for easier interpretation in all tables and figures.





**Supplementary Figure S4.** Scatter plots showing relationship between cross-sectional MTLTau, cross-sectional and longitudinal structural MRI measures in posterior hippocampus and BA35 as well as longitudinal change rate of clinical dementia rating sum of boxes (CDRSB) in cognitively unimpaired (CU) older adults. See Figure 1 for biomarker abbreviations. Structural MRI, cognition and tau PET measures were color-coded in green, red and purple respectively for easier interpretation in all tables and figures.


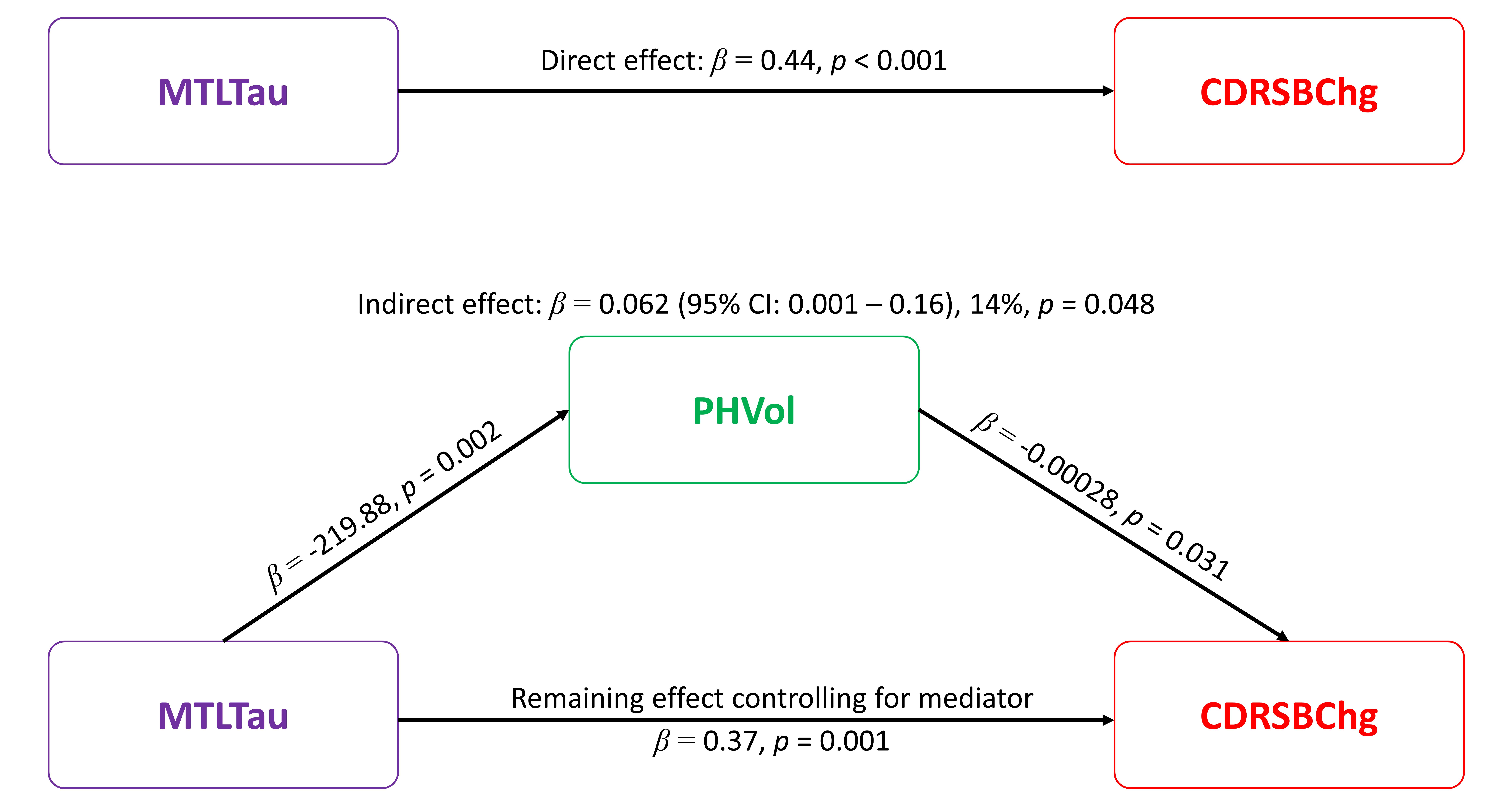


**Supplementary Figure S5.** Mediation analysis result investigating the mediation effect of cross-sectional posterior hippocampal volume (PHVol) for the correlation between medial temporal lobe tau burden (MTLTau) and longitudinal change rate of clinical dementia rating sum of boxes score (CDRSBChg). The result indicated PHVol partially mediated the correlation between MTLTau and CDRSBChg. See Figure 1 for biomarker abbreviations. Structural MRI, cognition and tau PET measures were color-coded in green, red and purple respectively for easier interpretation in all tables and figures.





**Supplementary Figure S6.** Receiver-operating characteristic (ROC) analyses for discriminating fast vs. slow progressors. Curves include models using none (yellow, null model with age, sex, education, APOE ɛ4 status, and follow-up time), only tau-based (purple), only MRI-based (green), only cognition-based (red) or all the (light blue) selected baseline cross-sectional biomarkers in Supplementary Table S1. Analyses were done in A- (first row) and A+ (second row) CU separately. Abbreviations: A+/A- = β-amyloid positive/negative; CU = cognitively unimpaired. See Figure 1 for biomarker abbreviations. Structural MRI, cognition and tau PET measures were color-coded in green, red and purple respectively for easier interpretation in all tables and figures.

**Supplementary Table S1.** Results of the stepwise linear mixed effect model analyses in the A+ and A- subgroups of cognitively unimpaired (CU) older adults for longitudinal atrophy [longitudinal posterior hippocampal volume (PHVol) and longitudinal ERC volume (ERCVol)] measurements. Variables fixed in the model: age, sex, education, APOE ɛ4 status and follow-up time. Baseline cross-sectional variables: MRI (green), tau PET (purple) and cognitive (red) biomarkers in Figure 1. See Figure 1 for biomarker abbreviations. Structural MRI, cognition and tau PET measures were color-coded in green, red and purple respectively for easier interpretation in all tables and figures.

| Dependent Variable | Group | Model statistics | Baseline measurements that are included in the model | |
| --- | --- | --- | --- | --- |
| Longitudinal PHVol | A- CU | N = 226  AIC = 399.8  R^2^ = 0.98  AUC = 0.76 | MTLTau  PHCThk | β = -0.02, *p* = 3.4x10^-5^  β = 0.01, *p* = 0.012 |
|  | A+ CU | N = 116  AIC = 109.1  R^2^ = 0.99  AUC = 0.86 | MTLTau  PHVol | β = -0.02, *p* = 1.5x10^-4^  β = 0.06, *p* = 6.1x10^-16^ |
| Longitudinal ERCVol | A- CU | N = 226  AIC = 396.5  R^2^ = 0.98  AUC = 0.70 | MTLTau | β = -0.01, *p* = 7.3x10^-4^ |
|  | A+ CU | N = 116  AIC = 154.3  R^2^ = 0.99  AUC = 0.77 | MTLTau | β = -0.02, *p* = 1.4x10^-3^ |

Note: AIC = Akaike information criterion; AUC = area under the curve; A+/A- = β-amyloid positive/negative; CU = cognitively unimpaired; ERC = entorhinal cortex.

**Supplementary Table S2.** Dependent variables, fixed effects, random effects and independent variables of linear mixed effect models used in the analysis of the predictive power of baseline biomarkers in disease progression (Section 2.5.3). See Figure 1 for biomarker abbreviations. Structural MRI, cognition and tau PET measures were color-coded in green, red and purple respectively for easier interpretation in all tables and figures.

| **Variable type** | **Measurements** | **Model inclusion** |
| --- | --- | --- |
| **Dependent variables** | - Longitudinal BA35Vol - Longitudinal DEL - Longitudinal CDRSB - Longitudinal PHVol - Longitudinal ERCVol | - One for each model in A- CU or A+ CU respectively |
| **Fixed effects** | - Age - Sex - Education - APOE ɛ4 status - Follow-up time | - Included in all the models |
| **Random effects** | - Random intercept for each participant - Random slope for time from baseline for each participant | - Included in all the models |
| **Independent variable candidates** | - Cross-sectional MRI   - AHVol   - PHVol   - ERCThk   - BA35Thk   - BA36Thk   - PHCThk - Cross-sectional MTLtau - Cross-sectional cognition   - DEL   - MMSE | - Included in the initial iterative search for all the models - Each measure was added in the model one at a time, together with its interaction with time from baseline |

**References.**

Arguillere, Sylvain, Emmanuel Trélat, Alain Trouvé, and Laurent Younes. 2015. 'Shape deformation analysis from the optimal control viewpoint', *Journal de mathématiques pures et appliquées*, 104: 139-78.

Bazin, P. L., and D. L. Pham. 2007. 'Topology correction of segmented medical images using a fast marching algorithm', *Comput Methods Programs Biomed*, 88: 182-90.

Charlier, Benjamin, Jean Feydy, Joan Alexis Glaunes, François-David Collin, and Ghislain Durif. 2021. 'Kernel operations on the GPU, with autodiff, without memory overflows', *Journal of Machine Learning Research*, 22: 1-6.

Charon, Nicolas, and Alain Trouvé. 2013. 'The varifold representation of nonoriented shapes for diffeomorphic registration', *SIAM journal on Imaging Sciences*, 6: 2547-80.

Fischl, B. 2012. 'FreeSurfer', *Neuroimage*, 62: 774-81.

Han, X., D. L. Pham, D. Tosun, M. E. Rettmann, C. Xu, and J. L. Prince. 2004. 'CRUISE: cortical reconstruction using implicit surface evolution', *Neuroimage*, 23: 997-1012.

Huntenburg, J. M., C. J. Steele, and P. L. Bazin. 2018. 'Nighres: processing tools for high-resolution neuroimaging', *Gigascience*, 7.

Isensee, F., P. F. Jaeger, S. A. A. Kohl, J. Petersen, and K. H. Maier-Hein. 2021. 'nnU-Net: a self-configuring method for deep learning-based biomedical image segmentation', *Nat Methods*, 18: 203-11.

Miller, M. I., A. Trouve, and L. Younes. 2006. 'Geodesic Shooting for Computational Anatomy', *J Math Imaging Vis*, 24: 209-28.

Tosun, D., M. E. Rettmann, X. Han, X. Tao, C. Xu, S. M. Resnick, D. L. Pham, and J. L. Prince. 2004. 'Cortical surface segmentation and mapping', *Neuroimage*, 23 Suppl 1: S108-18.

Yushkevich, Paul A, Long Xie, Laura EM Wisse, Mengjin Dong, Sadhana Ravikumar, Ranjit Ittyerah, Robin de Flores, Sandhitsu R Das, and David A Wolk. 2023. "Mapping Medial Temporal Lobe Longitudinal Change in Preclinical Alzheimer’s Disease." In *Alzheimer's & Dementia*, e081898.
